# Predictive potential of patient-specific immunological characteristics in solid cancers: circulating monocytes, myeloid-derived suppressor cells, T cells, and the T-cell receptor repertoire

**DOI:** 10.1016/j.esmoop.2025.105939

**Published:** 2025-12-31

**Authors:** C. Zierfuss, B. Niederdorfer, B. Fendl, K. Syböck, J. Schedl, L. Kohl, G. Heller, E. Tomasich, J.M. Berger, V. Sunder-Plassmann, M. Kleinberger, L. Gottmann, M. Korpan, A.M. Starzer, I. Solano Henao, J. Fürst, J. Wolfsberg, M. Grohmannova, N. Dobrovits, C. Ay, N. Vladic, J. Furtner, M. Preusser, A.S. Berghoff

**Affiliations:** 1 Division of Oncology, Department of Medicine I, Medical University of Vienna, Vienna, Austria; 2 Christian Doppler Laboratory for Personalized Immunotherapy, Department of Medicine I, Medical University of Vienna, Vienna, Austria; 3 Division of Hematology and Hemostaseology, Department of Medicine I, Medical University of Vienna, Vienna, Austria; 4 Research Center for Medical Image Analysis and Artificial Intelligence, Faculty of Medicine and Dentistry, Danube Private University, Krems, Austria

**Keywords:** immune checkpoint inhibition, patient-specific biomarker, solid cancer, monocytes, myeloid-derived suppressor cells, T-cell receptor repertoire

## Abstract

**Background:**

Response prediction to immune checkpoint inhibitors (ICIs) relies on tumor-specific biomarkers, while patient-specific characteristics are underrepresented. Therefore, we explored patient-specific immunological characteristics, including peripheral monocytes, myeloid-derived suppressor cells (MDSCs), T cells, and the T-cell receptor (TCR) repertoire, to investigate associations with survival and therapy response.

**Patients and methods:**

Patients with solid tumors were prospectively recruited to explore the association of absolute lymphocyte and monocyte counts, leukocyte-to-lymphocyte ratio (LLR), and monocyte-to-lymphocyte ratio (MLR) with overall survival (OS). Monocytes, MDSCs, T cells, and the TCR repertoire were characterized before therapy start (baseline) and, if available, at first radiological restaging (follow-up). We analyzed their association with radiological therapy response using current guidelines, OS, and progression-free survival (PFS).

**Results:**

A total of 1063 patients were included. High LLR [≥3.92; hazard ratio (HR) 1.48, 95% confidence interval (CI) 1.21-1.82, *P* < 0.001] and high MLR (≥0.31; HR 1.43, 95% CI 1.16-1.76, *P* < 0.001) at baseline were associated with worse OS. In 108 patients, high non-classical monocytes (NCMs) at baseline were linked to improved PFS (≥8.94%; HR 0.41, 95% CI 0.18-0.91, *P* = 0.03), indicating a prognostic effect independent of therapy type. High NCMs at baseline were associated with response to chemotherapy (*P* = 0.03), but not to ICI therapy (*P* = 0.47). Longitudinal analyses revealed an increase in intermediate monocytes (IMs) among ICI responders compared with ICI non-responders (*P* = 0.01). IMs were unaltered in chemotherapy-treated patients (*P* = 0.69). The TCR repertoire, T cells, and MDSCs revealed no differences between responders and non-responders, regardless of therapy.

**Conclusions:**

In our study, monocyte subsets were associated with survival and therapy response. These results highlight the potential of monocyte subsets to serve as patient-specific, tumor-agnostic biomarkers that may help predict therapy response.

## Highlights

• IMs and NCMs were associated with survival and therapy response to checkpoint inhibition.
• TCR diversity, T cells, and MDSCs were not linked to patient outcomes.
• Longitudinal monitoring of peripheral blood monocyte subsets might serve as a patient-specific, tumor-agnostic biomarker.

## Introduction

Current predictive biomarkers for immune checkpoint inhibitors (ICIs) include tumor mutational burden, programmed death-ligand 1 expression on tumor cells, and tumor-infiltrating lymphocytes,^1^ without accounting for patient-specific immunological characteristics. We therefore studied patient-specific properties that reflect individual immunological capacity, aiming to extend existing biomarkers and eventually improve therapy response prediction.

T cells, especially CD8^+^ T cells, are the primary effectors of ICI-based therapy.^2^ Therefore, peripheral T-cell composition and T-cell receptor (TCR) diversity have been proposed as minimally invasive, patient-specific liquid biomarkers, but associations with treatment outcomes remain inconsistent.^3^, ^4^, ^5^, ^6^, ^7^, ^8^, ^9^

Circulating monocytes, key components of the innate immune system, express human leukocyte antigen (HLA)-DR and are subdivided into classical (CMs; CD14^++^CD16^−^), intermediate (IMs; CD14^++^CD16^+^), and non-classical monocytes (NCMs; CD14^+^CD16^++^).^10^ Recently, monocyte subsets were shown to be associated with therapy response, survival, and resistance to ICI by suppressing peripheral T cells, highlighting the relevance of studying T cells in conjunction with monocytes.^11^, ^12^, ^13^

Similarly, myeloid-derived suppressor cells (MDSCs), heterogeneous cells with immunosuppressive skills arising from tumor-altered myelopoiesis, are characterized as CD33^+^, CD11b^+^, and HLA-DR^low/−^ and can be further classified into monocytic (M-MDSCs; CD14^+^CD15^−^), polymorphonuclear (PMN-MDSCs; CD14^−^CD15^+^), and early-stage MDSCs (CD14^−^CD15^−^).^14^^,^^15^ Total MDSCs have frequently been reported to be associated with resistance to ICI and poor clinical outcomes in patients with solid tumors.^14^^,^^16^, ^17^, ^18^

This study aimed to shed light on patient-specific characteristics that reflect the patient’s immunological ability to respond to cancer therapy. We recruited a large cancer patient cohort, including different cancer entities and treatment strategies, and analyzed various immune cell subtypes within the same patients to explore their tumor-agnostic biomarker potential.

## Patients and methods

### Patient selection, recruitment, and data collection

Patients were prospectively enrolled in the Biobanking Program of the Clinical Division of Oncology at the Medical University of Vienna between March 2019 and March 2025 (Supplementary Table S1, available at https://doi.org/10.1016/j.esmoop.2025.105939). Inclusion criteria were age ≥18 years, histologically confirmed solid tumor, and stage IV disease. Patients were included irrespective of tumor entity, prior treatments, or subsequent therapies, to represent the heterogeneity of patients treated at our institution.

In-depth immunological analyses were carried out in subsets of patients: monocyte and MDSC subsets in 108 patients, and T cells and their TCR repertoire in 84 patients (Supplementary Tables S2 and S3, available at https://doi.org/10.1016/j.esmoop.2025.105939). Additional inclusion restrictions were no prior ICI therapy, no radiotherapy within 6 weeks before inclusion, no steroid or granulocyte colony-stimulating factor treatment at the time of the first blood draw, and no (auto-)immunological disease or immune-modulating therapies. Monocyte and MDSC subset data are available for all patients with T-cell and TCR repertoire data, whereas the discrepancy in sample sizes reflects technical limitations rather than selection bias. Blood was drawn before treatment initiation (therapy-naive patients) or at therapy switch (pretreated patients) and, if available, after ∼3 months at the first radiological response assessment, referred to as baseline and follow-up, respectively. This longitudinal sampling approach enabled an assessment of dynamic changes related to cancer treatment and therapy response. Patients either received ICIs alone, ICIs combined with chemotherapy, chemotherapy alone, or chemotherapy combined with targeted therapy. For our analyses, the cohort was divided into two groups: patients receiving ICI-based therapy (ICIs alone or with chemotherapy) and those receiving chemotherapy-based therapy (chemotherapy alone or with targeted therapy), later referred to as ICI and chemotherapy, respectively.

The study was approved by the Ethics Committee of the Medical University of Vienna (approval numbers: 1164/2019, 1966/2021, and 2054/2021) and carried out according to the Declaration of Helsinki and its amendments. Written informed consent was obtained from patients before inclusion. Patients were treated according to the guidelines of Good Clinical Practice, and enrollment in this study did not influence therapy choice.

### Assessment of absolute blood cell counts

Conventional laboratory parameters, including absolute lymphocyte and monocyte cell counts, the leukocyte-to-lymphocyte ratio (LLR), the monocyte-to-lymphocyte ratio (MLR), the neutrophil-to-lymphocyte ratio (NLR), C-reactive protein (CRP), and lactate dehydrogenase (LDH) activity were extracted from differential blood count data obtained during clinical routine blood draws.

### Whole blood staining for monocyte and MDSC characterization

Blood samples were processed within 2 hours after collection (Supplementary Figure S1, available at https://doi.org/10.1016/j.esmoop.2025.105939). Briefly, 200 μl of sodium-heparin-anticoagulated blood was incubated with true-stain monocyte blocker (BioLegend, San Diego, CA). An antibody cocktail (Supplementary Table S4, available at https://doi.org/10.1016/j.esmoop.2025.105939) targeting CD3, CD11b, CD14, CD15, CD16, CD19, CD33, CD45, CD56, CD66b, HLA-DR, and 6-Sulfo LacNAc (slan) was prepared in stain buffer and brilliant stain buffer (both BD Biosciences, San Jose, CA), added, and incubated for 15 min. Next, erythrocytes were lysed with BD FACS lysing solution (BD Biosciences), washed with phosphate-buffered saline (PBS; Life Technologies, Paisley, UK), fixed with 1% paraformaldehyde (Boster Biological Technology, Pleasanton, CA), washed again, and resuspended in PBS. Acquisition was carried out on a Cytek Aurora (Cytek Biosciences, Fremont, CA) after carrying out the daily quality control to adjust for day-to-day instrument variability. Analysis was conducted using Kaluza Analysis Software (version 2.2; Beckman Coulter, Brea, CA). Sample measurement and analysis were carried out blinded, since the response status was unknown at these time points. The gating strategy is summarized in Supplementary Figure S2, available at https://doi.org/10.1016/j.esmoop.2025.105939. A whole blood approach was used to avoid loss of PMN-MDSCs due to their granulocytic phenotype^19^ (Supplementary Figure S3, available at https://doi.org/10.1016/j.esmoop.2025.105939).

### Isolation of PBMCs for T-cell characterization

Blood samples were processed within 2 hours after blood draw (Supplementary Figure S1, available at https://doi.org/10.1016/j.esmoop.2025.105939). Briefly, peripheral blood mononuclear cells (PBMCs) were isolated from EDTA-anticoagulated whole blood by density gradient centrifugation using Ficoll Paque PLUS (Cytiva, Marlborough, MA). PBMCs were collected and washed with PBS. Cells were stained for 15 min using antibodies targeting CD3, CD4, CD8, CD25, CD45, and CD45RO (Supplementary Table S5, available at https://doi.org/10.1016/j.esmoop.2025.105939). Cells were fixed with 1% paraformaldehyde, washed, and resuspended in PBS. Acquisition was carried out on a CytoFLEX S flow cytometer (Beckman Coulter) after carrying out the daily quality control to adjust for day-to-day instrument variability. Analysis was conducted using Kaluza Analysis Software. Sample measurement and analysis were carried out blinded, since the response status was unknown at these time points. The gating strategy is summarized in Supplementary Figure S4, available at https://doi.org/10.1016/j.esmoop.2025.105939.

### RNA isolation and TCR sequencing

RNA was isolated from PBMCs using the Monarch Total RNA Miniprep Kit with DNase I treatment (New England Biolabs, Ipswich, MA) and stored at −80°C. Bulk RNA sequencing was carried out by the Biomedical Sequencing Facility at CeMM Research Center for Molecular Medicine of the Austrian Academy of Sciences (Vienna, Austria) using the NEBNext Immune Sequencing Kit Human (New England Biolabs). High-throughput paired-end sequencing was carried out using the MiSeq platform (Illumina, San Diego, CA).

Raw sequencing data were processed using MiXCR (v4.7.0).^20^ Only duplicate reads with distinct unique molecular identifiers were further analyzed. Reads were aligned to a reference database containing V-, D-, and J-gene segments, and clonotypes were assembled, generating clonotype tables. Possible PCR- and sequencing-based errors were corrected. Clonotype tables were further analyzed using immunarch (v0.9.1)^21^ and vegan (v2.6-6.1)^22^ packages in R (V4.4.1, R Foundation for Statistical Computing, Vienna, Austria). TCR repertoires were described using the clonality index,^23^ diversity 50 (DV50) index,^24^ and effective number of clonotypes^25^ (see ‘Glossary’ section in the Supplementary Material, available at https://doi.org/10.1016/j.esmoop.2025.105939). Changes in indices were defined as [index (follow-up) – index (baseline)]/index (baseline).^26^

### Radiological therapy response assessment

Depending on the treatment regimen, patients underwent at least three treatment cycles before an independent, board-certified radiologist evaluated treatment response. Response was assessed according to the current guidelines of RECIST v1.1^27^ for patients receiving chemotherapy, and immune RECIST^28^ for patients receiving ICI-based therapy. We used two distinct endpoints: response at first radiological restaging (response at first follow-up) for analyses of longitudinal changes in variables between baseline and first follow-up, as well as the best response recorded from treatment start until disease progression during the whole treatment line (best overall response) for analyses of baseline variables. Successful response (responders) was defined as (immune) complete response or (immune) partial response, while lack of response (non-responders) was classified as (immune) stable disease or (immune) progressive disease. Patients who died before the first radiological response assessment due to clinically progressive disease were defined as non-responders. Dichotomized outcomes (responders versus non-responders) were used for both endpoints.

### Survival analysis

Overall survival (OS) and progression-free survival (PFS) were defined as the time from first treatment initiation (therapy-naive patients) or first therapy application at therapy switch (pretreated patients) until death or disease progression. Patients who were alive or who had not progressed by the time of data cut-off (8th January 2025) were censored at their last follow-up visit.

### Statistical analysis

Analyses were conducted using Prism (v10.4.0; GraphPad Software Inc., San Diego, CA) and R (v4.4.1, R Foundation for Statistical Computing). Normality was tested using the Shapiro–Wilk test. To assess the influence of age, we divided our cohort into quartiles based on the patient’s age at study inclusion. Differences between groups were assessed using the Mann–Whitney *U* test or Kruskal–Wallis test. For survival analyses, variables were dichotomized as high or low using either the first quartile (Q1) or the median (Q2) as cut-offs, specified for each experiment. Survival was evaluated using Kaplan–Meier plots and log-rank tests. Entity-stratified Cox proportional hazards models were used to explore prognostic effects of tested variables, with cancer entity included as a stratification factor to account for survival heterogeneity across cancer types. Predictive effects were assessed by including interaction terms between the tested variables and type of therapy. Clinically relevant covariates [sex, age, pretreatment status, type of therapy, Eastern Cooperative Oncology Group (ECOG) performance status, number of metastases, LDH activity] were included. Results are reported as hazard ratios (HRs) with 95% confidence intervals (CIs). Correlation analyses were carried out using Spearman’s rank correlation coefficients. Significance was defined as two-tailed *P* ≤ 0.05 (^∗^*P* < 0.05, ^∗∗^*P* < 0.01, ^∗∗∗^*P* < 0.001). No multiple-testing correction was applied due to the exploratory study design.^29^

## Results

### MLR and LLR are predictive of OS, irrespective of the primary tumor type

**Figure 1:**
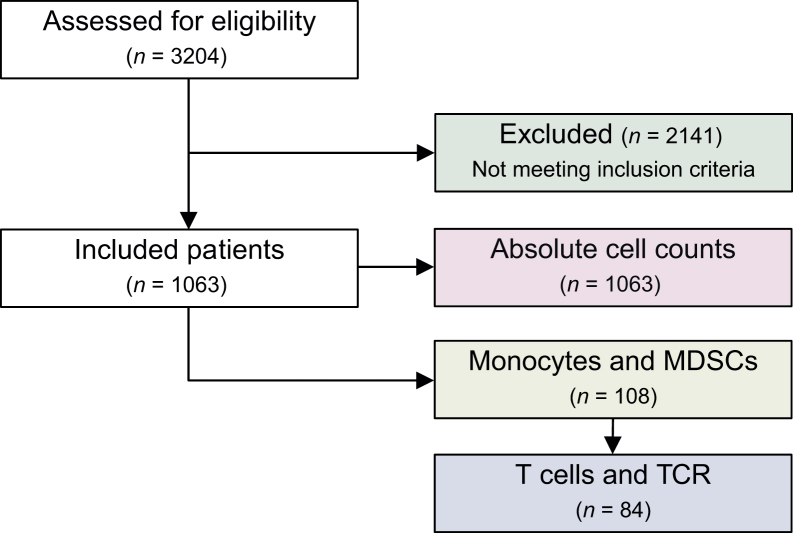
**CONSORT diagram providing an overview of included patients for the analysis of absolute cell counts, monocyte and MDSC subsets, as well as T cells and their receptor repertoires.** CONSORT, Consolidated Standards of Reporting Trials; MDSC, myeloid-derived suppressor cell; TCR, T-cell receptor.

Firstly, we assessed the association of conventional laboratory parameters with OS in our cohort, including peripheral lymphocyte and monocyte counts, LLR, and MLR and included 1063 patients with advanced cancer who were treated with systemic therapy (Figure 1). The median age at enrollment was 64.0 years [standard deviation (SD) ±12.6], and 53.2% of patients were male. Patients most frequently presented with lung cancer (23.0%), pancreatic and biliary tract cancer (18.3%), and lower gastrointestinal tract cancer (12.7%). The median OS in the cohort was 9.0 months, with 37.6% of patients alive at the cut-off date. Patient characteristics are displayed in Supplementary Table S1, available at https://doi.org/10.1016/j.esmoop.2025.105939.

**Figure 2:**
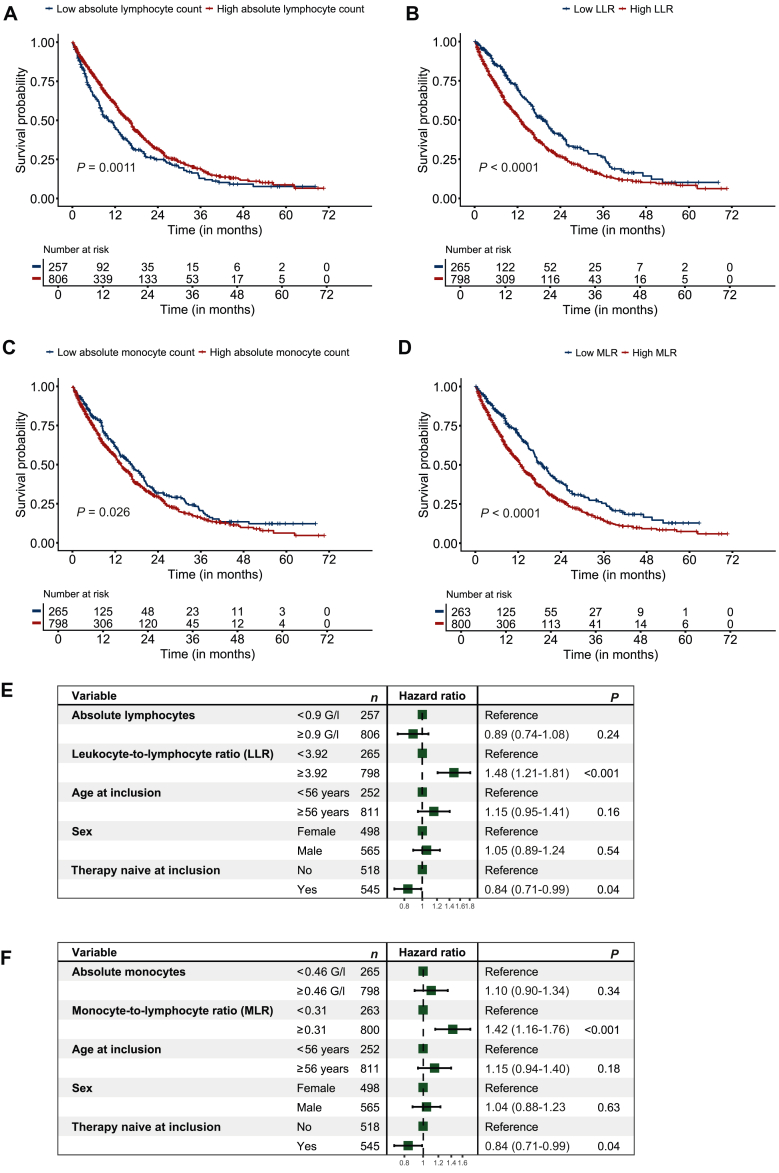
**Association of baseline lymphocyte- and monocyte-based indices with overall survival.** Kaplan–Meier plots displaying the overall survival probability for patients with high or low (A) absolute lymphocyte count, (B) leukocyte-to-lymphocyte ratio (LLR), (C) absolute monocyte count, and (D) monocyte-to-lymphocyte ratio (MLR) at baseline. Variables were dichotomized using the first quartile (Q1) as cut-off. Survival differences were assessed using log-rank test. Forest plot displaying results from an entity-stratified multivariable Cox proportional hazards regression model, assessing the effect of (E) absolute lymphocyte count and LLR, and (F) absolute monocyte count and MLR on OS. Hazard ratios (HRs) with 95% confidence intervals (CIs) are given. HR < 1 indicates improved survival, and HR > 1 indicates worse survival. *n* = 1063.

When using the first quartile as cut-off, we observed prolonged OS in patients with high absolute lymphocyte counts (≥0.9 G/l; *P* < 0.001), low LLR (<3.92; *P* < 0.001), low absolute monocyte counts (<0.46 G/l; *P* < 0.05), and low MLR (<0.31; *P* < 0.001) at baseline (Figure 2A-D), as determined by Kaplan–Meier analyses. In subsequent entity-stratified multivariable Cox proportional hazards regression models, high LLR (≥3.92; HR 1.48, 95% CI 1.21-1.82, *P* < 0.001) and high MLR (≥0.31; HR 1.43, 95% CI 1.16-1.76, *P* < 0.001) remained significant predictors of worse OS (Figure 2E and F). Therapy-naive patients at study inclusion presented with improved OS within both models (HR 0.83, 95% CI 0.70-0.98, *P* = 0.03), while sex and age at inclusion had no impact.

### NCMs are associated with patient survival

Next, we conducted a comprehensive analysis of monocyte and MDSC subsets in 108 out of 1063 recruited patients, with 79 available longitudinal samples. In addition, we carried out an in-depth characterization of T cells and their receptor repertoire in 84 out of 108 recruited patients, with 73 available follow-up samples.

**Figure 3:**
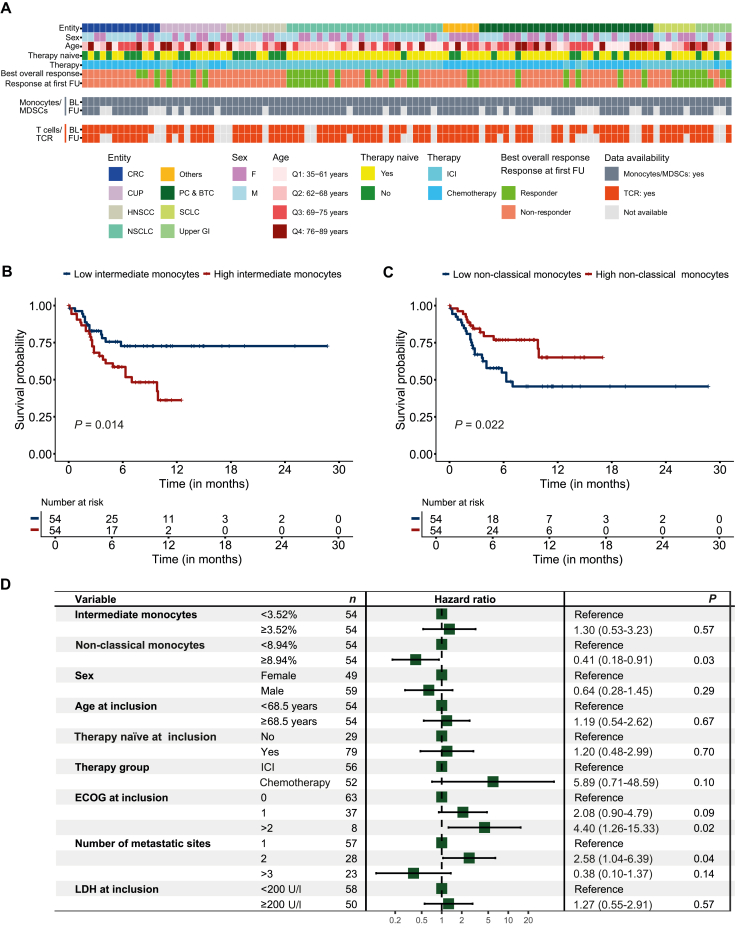
**Clinical annotation and association of baseline monocyte subsets with progression-free survival.** (A) Clinical annotation heatmap of enrolled patients displaying demographics and availability of flow cytometry and TCR sequencing data. (B, C) Kaplan–Meier plots displaying the progression-free survival probability for patients with high or low (B) intermediate (IMs) and (C) non-classical monocytes (NCMs) at baseline. Variables were dichotomized using the median as cut-off. Survival differences were assessed using the log-rank test. (D) Forest plot displaying results from an entity-stratified multivariable Cox proportional hazards regression model assessing the effect of IMs and NCMs on progression-free survival, while including clinically relevant covariates. Hazard ratios (HRs) with 95% confidence intervals (CIs) are given. HR < 1 indicates improved survival, and HR > 1 indicates worse survival. *n* = 108. BL, baseline; CRC, colorectal cancer; CUP, cancer of unknown primary; ECOG, Eastern Cooperative Oncology Group; F, female; FU, follow-up; HNSCC, head and neck squamous-cell carcinoma; ICI, immune checkpoint inhibition; M, male; NA, not available; NSCLC, non-small-cell lung cancer; PC & BTC, pancreatic cancer and biliary tract cancer; SCLC, small-cell lung cancer; upper GI, upper gastrointestinal tract cancer.

In the subcohort of 108 patients, the median age at enrollment was 68.5 years (SD ±11.4), and 54.5% of patients were male. The most frequently represented entities were lung cancer (30.6%), pancreatic and biliary tract cancer (26.9%), and colorectal cancer (12.0%). Patients were either treated with ICI- (51.9%) or chemotherapy-based therapy (48.1%). The median PFS and OS were 4.9 and 8.0 months, respectively, with 64.8% of patients alive at the cut-off date. The minimum observation period was 1.8 months. Patient characteristics were comparable between the two cohorts, including 108 and 84 patients, respectively. Patient characteristics are displayed in Figure 3A and Supplementary Tables S2 and S3, available at https://doi.org/10.1016/j.esmoop.2025.105939.

When using the median as cut-off, Kaplan–Meier analyses revealed significant differences in patient survival with prolonged PFS and OS observed in patients with low IMs (<3.52%; PFS *P* = 0.014; OS *P* = 0.005) and high NCMs (≥8.94%; PFS *P* = 0.022; OS *P* = 0.048) (Figure 3B and C, Supplementary Figure S5A and B, available at https://doi.org/10.1016/j.esmoop.2025.105939). In a subsequent entity-stratified multivariable Cox proportional hazards regression model, high NCMs at baseline were associated with better PFS (HR 0.41, 95% CI 0.18-0.91, *P* = 0.03), but not OS (Figure 3D), indicating a prognostic effect. Low IMs at baseline were not associated with improved survival (Figure 3D, Supplementary Figure S5C, available at https://doi.org/10.1016/j.esmoop.2025.105939). IM and NCM levels were independent of the inflammation markers CRP and NLR, and an additional Cox proportional hazards regression model validated an independent protective effect of NCMs on PFS (Supplementary Figure S6, available at https://doi.org/10.1016/j.esmoop.2025.105939). Interaction analyses between IMs, NCMs, and the therapy group revealed no differences (all *P* > 0.05), indicating no predictive effect of IMs and NCMs for therapy-specific outcomes (Supplementary Figures S7 and S8, available at https://doi.org/10.1016/j.esmoop.2025.105939). Sex, age, pretreatment, therapy group, and LDH activity had no impact, while ECOG >2 and more than two metastatic sites were predictive of survival (Figure 3D, Supplementary Figure S5C, available at https://doi.org/10.1016/j.esmoop.2025.105939). T cells and their TCR repertoire, total monocytes, CMs, total MDSCs, and their subsets (Supplementary Figures S9-S14, available at https://doi.org/10.1016/j.esmoop.2025.105939) did not associate with survival.

### Peripheral T-cell distribution and receptor repertoire are comparable between responders and non-responders

Although T cells and their receptor repertoire were not associated with patient survival, we sought to evaluate whether they are linked to therapy response. Firstly, we examined the potential impact of patient-intrinsic, tumor-specific, and pretreatment-related factors at baseline. We detected lower CD3^+^ T cells (*P* = 0.01) and CD4^+^ T cells (*P* = 0.04) and higher CD25^+^ T_regs_ (*P* < 0.001) and CD4^+^CD45RO^+^ T cells (*P* < 0.01) in male patients. Female patients presented with a less clonal (*P* = 0.027) and more diverse TCR repertoire (DV50 *P* < 0.001; number of clonotypes *P* < 0.01) than male patients. CD3^+^ T cells were highest in patients aged between 62 and 68 years (Q2 versus Q1 *P* = 0.01; Q2 versus Q3 *P* < 0.01; Q2 versus Q4 *P* < 0.01), whereas age did not affect the TCR repertoire (*P* > 0.05). The tumor type, pretreatment, and systemic cancer treatment before study inclusion did not affect peripheral T-cell and TCR repertoire composition (*P* > 0.05; Supplementary Figures S15 and S16, available at https://doi.org/10.1016/j.esmoop.2025.105939).

**Figure 4:**
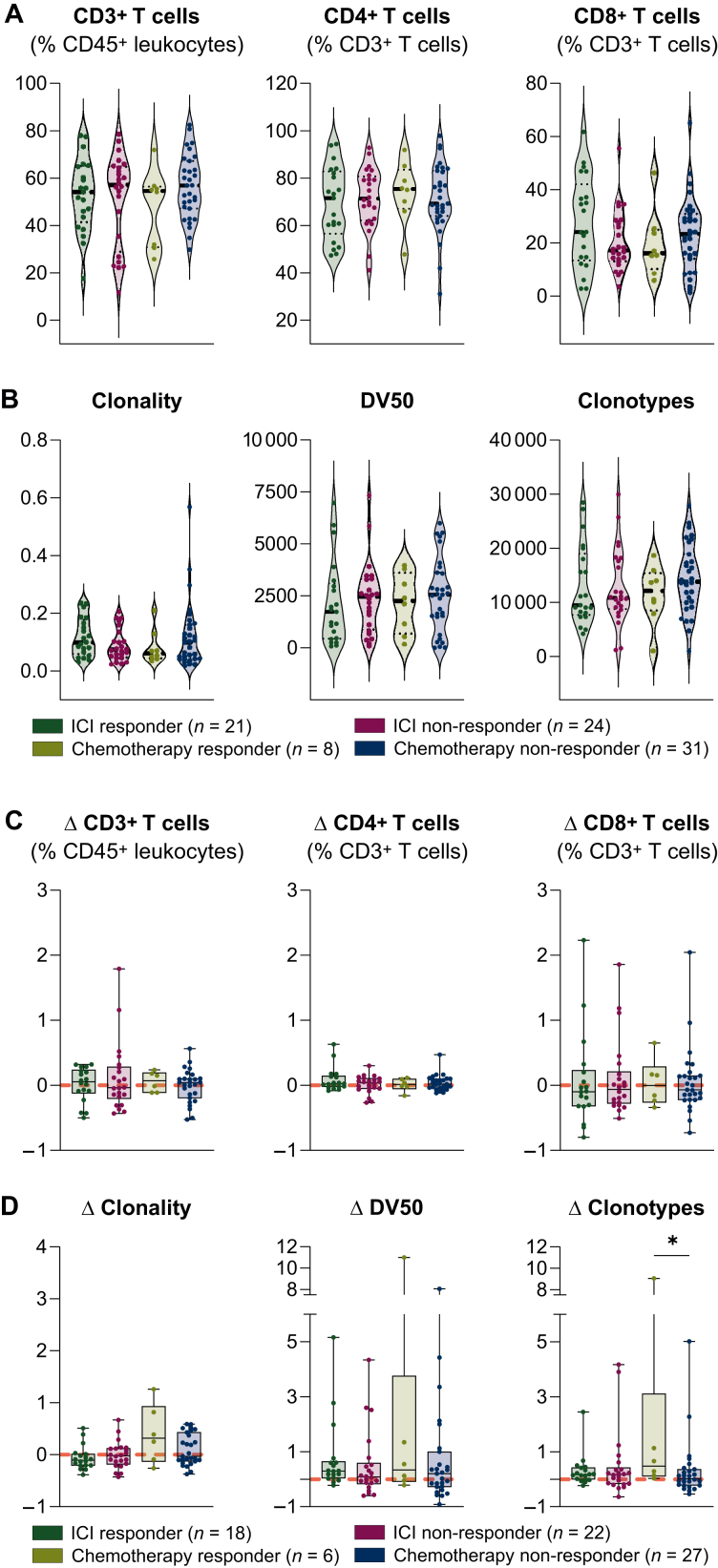
**Association of T cell subsets and TCR repertoire characteristics with therapy response.** Violin plots displaying differences at baseline in (A) CD3^+^, CD4^+^, and CD8^+^ T-cell distribution, and (B) TCR clonality and diversity among ICI and chemotherapy responders and non-responders. Dashed lines represent the median, and dotted lines represent the 25^th^ and 75^th^ percentiles. Boxplots present dynamic changes between baseline and first follow-up as delta in (C) and (D), respectively. T-cell subsets are displayed as a percentage of their respective parent population. Unpaired Mann–Whitney *U* test was used to compare differences between groups. DV50, diversity 50; ICI, immune checkpoint inhibitor. ^∗^*P* < 0.05.

Next, we evaluated the associations between T-cell composition and TCR characteristics and therapy response. Overall, baseline T-cell composition (Figure 4A, Supplementary Figure S17, available at https://doi.org/10.1016/j.esmoop.2025.105939) and TCR metrics (Figure 4B) showed no association with the likelihood of therapy response at first follow-up (*P* > 0.05). Furthermore, no dynamic changes in peripheral T-cell composition between baseline and follow-up were observed between responders and non-responders (*P* > 0.05; Figure 4C, Supplementary Figure S17, available at https://doi.org/10.1016/j.esmoop.2025.105939). Dynamic changes in TCR repertoire clonality were comparable between ICI responders and ICI non-responders (*P* > 0.05; Figure 4D). However, we observed a significantly higher number of clonotypes at first follow-up in chemotherapy responders (*P* = 0.04), while the clonotype count remained unaltered in chemotherapy non-responders (Figure 4D).

### IMs increase in immunotherapy responders

After observing an association between high NCMs and improved PFS, we aimed to evaluate whether this might be linked to a better treatment response. We studied patient-, pretreatment-related, and tumor-specific effects on monocytes and MDSCs at baseline and found higher total monocytes in male and pretreated patients (both *P* < 0.01). M-MDSCs were highest in both younger (<62 years) and older (>75 years) patients (Q1 versus Q2 *P* = 0.02; Q4 versus Q2 *P* < 0.01; Q4 versus Q3 *P* = 0.02). Cancer entity did not affect subsets (*P* > 0.05; Supplementary Figure S18, available at https://doi.org/10.1016/j.esmoop.2025.105939).

**Figure 5:**
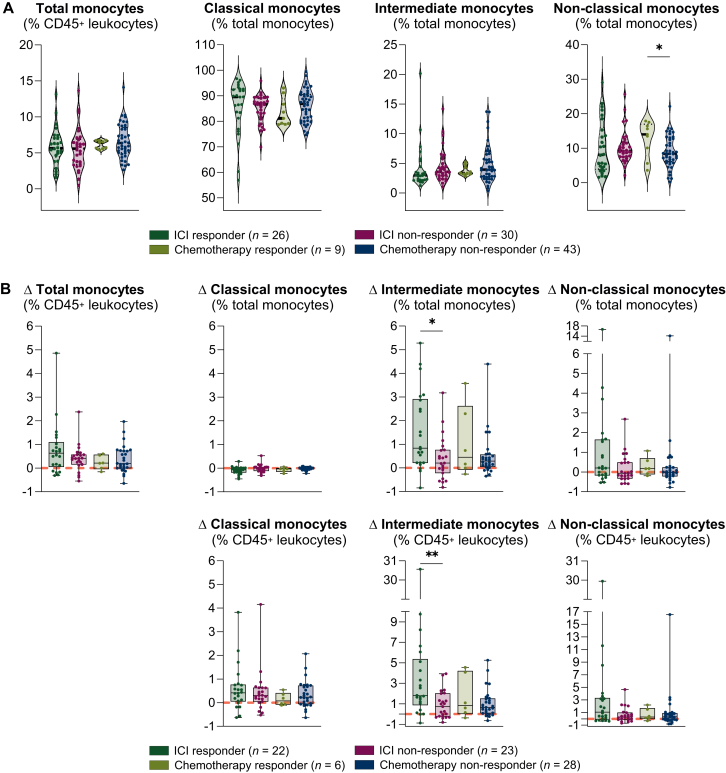
**Association of monocyte subsets with therapy response.** Violin plots displaying differences at baseline in (A) total, classical, intermediate, and non-classical monocyte distribution among ICI and chemotherapy responders and non-responders. Dashed lines represent the median, and dotted lines represent the 25^th^ and 75^th^ percentiles. Dynamic changes between baseline and first follow-up are presented as delta in the boxplots (B). Monocyte subsets are displayed as a percentage of total monocytes and percentage of CD45^+^ leukocytes. Unpaired Mann–Whitney *U* test was used to compare differences between groups. ICI, immune checkpoint inhibitor. ^∗^*P* < 0.05, ^∗∗^*P* < 0.01.

When studying baseline metrics, we did not detect any differences regarding the likelihood of therapy response at first follow-up in total monocytes, CMs, IMs (Figure 5A), and MDSCs (all *P* > 0.05; Supplementary Figure S19, available at https://doi.org/10.1016/j.esmoop.2025.105939). However, higher NCMs at baseline were associated with subsequent response in patients treated with chemotherapy compared with chemotherapy non-responders (*P* = 0.03; Figure 5A). No association of baseline NCMs with response was observed in ICI-treated patients (*P* > 0.05; Figure 5A). When assessing dynamic changes between baseline and first follow-up, we did not observe any differences in total monocytes, CMs, NCMs (Figure 5B), or MDSCs (all *P* > 0.05; Supplementary Figure S19, available at https://doi.org/10.1016/j.esmoop.2025.105939). In contrast, IM significantly increased in ICI responders compared with ICI non-responders (*P* = 0.01; Figure 5B), indicating a therapy-associated immune modulation. This increase was also detectable when gating IMs from CD45^+^ leukocytes, indicating an absolute increase (*P* = 0.007; Figure 5B). Simultaneously, CMs tended to decrease in ICI-treated responders compared with ICI-treated non-responders (*P* = 0.06; Figure 5B). These alterations were not observed in patients treated with chemotherapy (*P* > 0.05; Figure 5B).

## Discussion

Monocyte subsets were associated with PFS and therapy response in our study, highlighting their tumor-agnostic importance. While lymphocyte count was also associated with survival, detailed T-cell features, including T-cell subsets and TCR repertoire metrics, did not correlate with response or survival. These findings underscore the potential of monocyte subsets as biomarkers and targets for novel immune-modulating therapies.

Patient-specific immunological characteristics, such as monocyte or lymphocyte counts, were previously proposed as tumor-agnostic biomarkers influencing both the clinical course and therapy response.^30^ Consistently, we observed an entity-, sex-, and age-independent association between monocyte and lymphocyte counts and survival prognosis. High lymphocyte levels have repeatedly been associated with favorable OS within the context of NLR,^31^ but LLR has been rarely addressed. Although one study reported an association of high LLR with worse OS in T-cell lymphoma,^32^ validation of LLR in a large cohort, including solid tumors like ours, has so far been missing. The observed low LLR may indicate successful lymphocyte activation, which can mediate effective antitumor responses and contribute to the observed improved survival. Supporting our hypothesis, *ex vivo* stimulation of patient-derived lymphocytes resulted in a significantly higher proportion of activated CD8^+^ T cells in ICI responders than in non-responders.^33^

An association between low MLR and improved survival has already been observed in several malignancies across retrospective studies,^34^^,^^35^ which we could confirm in our prospective cohort. We hypothesized that this link might be impacted by limited differentiation of circulating monocytes into tumor-associated macrophages (TAMs) within the tumor microenvironment (TME). In line with this, studies have linked the peripheral monocyte count to TAM count and density in the TME^36^^,^^37^ and increased monocyte infiltration is associated with poor prognosis.^38^ Further, we observed that high NCMs at baseline are linked to prolonged OS, independent of the treatment type, which is consistent with another study.^39^ Moreover, we found higher pretreatment levels of NCMs to be associated with the likelihood of chemotherapy response at first follow-up. While this has not been observed in other studies, we hypothesized that their patrolling behavior, in combination with their ability to clear dying cancer cells in the circulation and recruit natural killer cells, might contribute to successful chemotherapy response.^40^ Differences with other studies reporting higher NCMs in ICI non-responders^13^ might be explained by methodology, as we analyzed whole blood rather than PBMCs, allowing concomitant characterization of monocytes and MDSCs. Furthermore, we observed a dynamic increase of IM within ICI responders, which was undetectable in ICI non-responders, suggesting enhanced differentiation of CMs into IMs or limited migration of CMs into tissues. IMs may contribute to successful ICI therapy response by enhancing antibody-dependent cellular cytotoxicity.^41^ While comparable studies are yet missing, other studies repeatedly showed increased IMs in cancer patients.^42^^,^^43^ Overall, our findings support the crucial role of monocytes in generating effective, tumor-specific immune responses. In line with this, novel immune-modulating approaches focus on the combination of T-cell- and monocyte-directed therapies, intending to generate durable immune responses.^44^^,^^45^

We did not detect differences in T-cell subsets or in key TCR repertoire metrics, including clonality, DV50, and effective clonotype number. The similarity of TCR-related indices between responders and non-responders to either ICI or chemotherapy suggests that treatment-associated alterations in the peripheral TCR repertoire are minimal in our cohort. So far, published data on TCR repertoires are conflicting, as some researchers reported associations with therapy response,^3^^,^^4^ while others observed no differences.^7^^,^^8^ Associations with response or survival are primarily observed in NSCLC patients,^3^^,^^4^ which might suggest an entity-dependent effect. However, we did not observe differences attributed to cancer entity in our cohort. Discrepancies might be caused by missing standardized guidelines, as some studies carry out TCR analyses on PBMCs,^3^, ^4^, ^5^, ^6^, ^7^, ^8^ while others conduct targeted CD8^+^^46^ or CD8^+^PD-1^+^ T-cell sequencing.^26^ While total PBMCs contain many non-tumor-specific TCRs, prior studies have shown associations with patient outcomes.^3^, ^4^, ^5^, ^6^^,^^24^^,^^26^ We focused on PBMCs for feasibility in clinical biomarker development and used commonly employed indices to characterize TCR diversity and clonality.^3^^,^^7^ Although we did not detect differences between responders and non-responders, our findings contribute to a more standardized approach.

When focusing on MDSCs, we observed no differences between responders and non-responders. Studies reported elevated M-MDSCs at baseline in non-responders^13^^,^^17^ and a correlation of low M-MDSCs with improved survival.^13^^,^^18^ In contrast, we observed a trend toward improved survival with higher M-MDSCs (*P* = 0.055), likely due to differences in study design, gating strategies, or cell isolation methods. Although efforts have been made to standardize MDSC characterization,^47^^,^^48^ there are still major differences. Whole blood analysis, as carried out here, may better reflect the *in vivo* composition, including PMN-MDSCs, which are lost during PBMC isolation.^19^ Moreover, PBMC isolation requires an additional processing step, which might introduce technical artifacts and has also been shown to lead to interinstitutional variability.^48^^,^^49^ Our data suggest that whole blood characterization of MDSCs is feasible and may provide a foundation for future studies, despite the absence of predictive effects in our cohort.

Some limitations need to be considered. The descriptive nature of our study does not allow conclusions regarding underlying biological mechanism. We omitted multiple-testing correction, which is why our results should be interpreted with caution, as some associations may not reflect true biological effects. Furthermore, the ICI therapy group includes patients receiving ICI alone and in combination with chemotherapy, since this is a prospective study that comes with small sample sizes in each subgroup, which also needs to be considered when interpreting our results. However, using a tumor-agnostic approach, such as our large patient cohort, may support the potential generalizability of our findings across various cancer types.

In conclusion, we found significant associations of monocyte subsets that may help predict therapy response in patients with solid cancers. These findings underscore the importance of patient-specific, tumor-agnostic immunological characteristics in evaluations, warranting further investigation.
