# Supplementary material for: Predictive potential of patient-specific immunological characteristics in solid cancers: circulating monocytes, myeloid-derived suppressor cells, T cells, and the T-cell receptor repertoire
Source: ESMO Open. 2025 Dec 31;11(1):105939. doi: 10.1016/j.esmoop.2025.105939 (PMC12804612; doi:10.1016/j.esmoop.2025.105939)
Supplement: Supplementary Material [file mmc1.docx]

## **Supplemental Material:**

## **“Predictive Potential of Patient-Specific Immunological Characteristics in Solid Cancers: Circulating Monocytes, Myeloid-Derived Suppressor Cells, T Cells, and the T Cell Receptor Repertoire”**

Carina Zierfuss^1,2^, Barbara Niederdorfer^1,2^, Birgit Fendl^1,2^, Katharina Syböck^1,2^, Julia Schedl^1,2^, Leonie Kohl^1,2^, Gerwin Heller^1^, Erwin Tomasich^1,2^, Julia Maria Berger^1,2^, Vincent Sunder-Plassmann^1,2^, Markus Kleinberger^1,2^, Lynn Gottmann^1,2^, Martin Korpan^1,2^, Angelika Martina Starzer^1,2^, Isabella Solano Henao^1^, Josef Fürst^1^, Johanna Wolfsberg^1^, Miriam Grohmannova^1^, Noah Dobrovits^1^, Cihan Ay^3^, Nikola Vladic^3^, Julia Furtner^2,4^, Matthias Preusser^1,2^, Anna Sophie Berghoff^1,2*^

1. Division of Oncology, Department of Medicine I, Medical University of Vienna, Vienna, Austria
2. Christian Doppler Laboratory for Personalized Immunotherapy, Department of Medicine I, Medical University of Vienna, Vienna, Austria
3. Division of Hematology and Hemostaseology, Department of Medicine I, Medical University of Vienna, Vienna, Austria
4. Research Center for Medical Image Analysis and Artificial Intelligence, Faculty of Medicine and Dentistry, Danube Private University, Krems, Austria

* Corresponding author:

Anna Sophie Berghoff, MD, PhD

Division of Oncology, Department of Medicine I

Medical University of Vienna

Waehringer Guertel 18-20, 1090 Vienna, Austria

Phone: +43 1 40400 44450

E-mail: anna.berghoff@meduniwien.ac.at

**Glossary**

Clonality: Evaluates clonal expansion and is defined as the probability that two independent sequences originate from the same clone. Clonality is inversely related to diversity (high clonality = low diversity).

Clonality Index: Pielou’s evenness measures clonal dominance, while 0 represents an uneven repertoire consisting of only one clonotype (high clonality) and 1 an even repertoire (low clonality). The Clonality Index is more frequently used to provide an intuitive interpretation and is defined as 1-Pielou’s evenness, where 0 represents an even (low clonality) and 1 an uneven repertoire (high clonality) (24).

Diversity: Reflects the number of different unique clonotypes (richness) and their abundance (evenness) (24).

DV50 Index: Represents the percentage of different clonotypes occupying 50% of the repertoire, whereas 0 indicates a repertoire dominated by a few different clonotypes (high clonality) and 1 an even clonotype distribution (low clonality) (25).

Effective Number of Clonotypes: The Shannon Index, also known as the Shannon Diversity Index or the Shannon-Wiener Index, measures diversity by accounting for richness and evenness, and ranges from 0 (no diversity) to higher values, depending on the sample complexity. The Shannon Effective Diversity, or Effective Number of Clonotypes, provides a more intuitive interpretation, as it converts the Shannon Index into the number of equally abundant clonotypes. High values indicate an even clonotype distribution with high diversity (26).

Evenness: Describes the distribution of the frequencies of unique clonotypes (24).

Richness: Number of unique TCR sequences (also referred to as ‘clonotypes’), which are generated by random V(D)J gene-segment recombination (4).

**Supplementary Tables**

**Supplementary Table 1:** Patient characteristics of the patient cohort for the evaluation of conventional laboratory parameters with OS (n = 1,063).

| **Patient characteristics** | | **n = 1,063** | **%** |
| --- | --- | --- | --- |
| **Sex** | Female | 498 | 46.8 |
|  | Male | 565 | 53.2 |
| **Age** | Median [years] | 64.0 | - |
|  | Range | 18-91 | - |
| **Entity** | Non-small cell lung cancer | 245 | 23.0 |
|  | Pancreatic and biliary tract cancer | 194 | 18.3 |
|  | Lower gastrointestinal tract cancer | 135 | 12.7 |
|  | Sarcoma | 99 | 9.3 |
|  | Head and neck squamous cell cancer | 88 | 8.3 |
|  | Upper gastrointestinal tract cancer | 74 | 7.0 |
|  | Breast cancer | 70 | 6.6 |
|  | Small cell lung cancer | 40 | 3.8 |
|  | Cancer of unknown primary | 34 | 3.2 |
|  | Urogenital tract cancer | 34 | 3.2 |
|  | Others (n < 4) | 32 | 3.0 |
|  | Neuroendocrine tumors | 18 | 1.7 |
| **Treatment** | Immune checkpoint inhibitor-based | 409 | 38.5 |
|  | Chemotherapy-based | 654 | 61.5 |
| **Previous treatment** | Yes | 518 | 48.7 |
|  | No | 545 | 51.3 |
| **Overall survival** | Median [months] | 9.0 | - |
|  | Range | 0.1-70.6 | - |

**Supplementary Table 2:** Patient characteristics of the patient cohort for monocyte/MDSC characterization (n = 108).

| **Patient characteristics** | | **n = 108** | **%** |
| --- | --- | --- | --- |
| **Sex** | Female | 49 | 45.4 |
|  | Male | 59 | 54.6 |
| **Age** | Median [years] | 68.5 | - |
|  | Range | 25-91 | - |
| **Entity** | Non-small cell lung cancer | 26 | 24.1 |
|  | Pancreatic and biliary tract cancer | 29 | 26.9 |
|  | Colorectal cancer | 13 | 12.0 |
|  | Cancer of unknown primary | 11 | 10.2 |
|  | Head and neck squamous cell cancer | 10 | 9.3 |
|  | Small cell lung cancer | 7 | 6.5 |
|  | Upper gastrointestinal tract cancer | 6 | 5.6 |
|  | Others (n < 4) | 6 | 5.6 |
| **Treatment** | Immune checkpoint inhibitor-based | 56 | 51.9 |
|  | Chemotherapy-based | 52 | 48.1 |
| **Previous treatment** | Yes | 29 | 26.9 |
|  | No | 79 | 73.1 |
| **ECOG performance status** | 0 | 63 | 58.3 |
|  | 1 | 37 | 34.3 |
|  | > 2 | 8 | 7.4 |
| **Number of metastatic sites** | 1 | 57 | 52.8 |
|  | 2 | 28 | 25.9 |
|  | > 3 | 23 | 21.3 |
| **Response (BOR)** | Responder | 35 | 32.4 |
|  | Non-responder | 73 | 67.6 |
| **Response (1^st^ follow-up)** | Responder | 31 | 28.7 |
|  | Non-responder | 77 | 71.3 |
| **Progression-free survival** | Median [months] | 4.9 | - |
|  | Range | 0-28.7 | - |
| **Overall survival** | Median [months] | 8.0 | - |
|  | Range | 0-28.7 | - |
| **Follow-up time** | Median [days] | 69 | - |
|  | Range | 42-190 | - |
|  | Available follow-up samples | 79 | 73.1 |

BOR = best overall response, ECOG = Eastern Cooperative Oncology Group, MDSC = myeloid-derived suppressor cell.

**Supplementary Table 3:** Patient characteristics of the patient cohort for T cell and TCR repertoire characterization (n = 84).

| **Patient characteristics** | | **n = 84** | **%** |
| --- | --- | --- | --- |
| **Sex** | Female | 36 | 42.9 |
|  | Male | 48 | 57.1 |
| **Age** | Median [years] | 68.5 | - |
|  | Range | 35-89 | - |
| **Entity** | Non-small cell lung cancer | 21 | 25.0 |
|  | Pancreatic and biliary tract cancer | 16 | 19.0 |
|  | Colorectal cancer | 12 | 14.3 |
|  | Others (n < 4) | 10 | 11.9 |
|  | Head and neck squamous cell cancer | 8 | 9.5 |
|  | Cancer of unknown primary | 7 | 8.3 |
|  | Small cell lung cancer | 6 | 7.1 |
|  | Upper gastrointestinal tract cancer | 4 | 4.8 |
| **Treatment** | Immune checkpoint inhibitor-based | 45 | 53.6 |
|  | Chemotherapy-based | 39 | 46.4 |
| **Previous treatment** | Yes | 26 | 31.0 |
|  | No | 58 | 69.0 |
| **ECOG performance status** | 0 | 53 | 63.1 |
|  | 1 | 27 | 32.1 |
|  | > 2 | 4 | 4.8 |
| **Number of metastatic sites** | 1 | 42 | 50.0 |
|  | 2 | 22 | 26.2 |
|  | > 3 | 20 | 23.8 |
| **Response (BOR)** | Responder | 29 | 34.5 |
|  | Non-responder | 55 | 65.5 |
| **Response (1^st^ follow-up)** | Responder | 26 | 31.0 |
|  | Non-responder | 58 | 69.0 |
| **Progression-free survival** | Median [months] | 5.2 | - |
|  | Range | 0-28.7 | - |
| **Overall survival** | Median [months] | 8.8 | - |
|  | Range | 0-28.7 | - |
| **Follow-up time** | Median [days] | 70 | - |
|  | Range | 42-190 | - |
|  | Available follow-up samples | 73 | 86.9 |

BOR = best overall response, ECOG = Eastern Cooperative Oncology Group, TCR = T cell receptor.

**Supplementary Table 4:** Antibodies and reagents used for monocyte and MDSC subset characterization.

| **Antibody/Reagent** | **Fluorochrome** | **Clone** | **Dilution** | **Manufacturer** | **Article number** |
| --- | --- | --- | --- | --- | --- |
| Brilliant stain buffer | - | - | 1:2 | BD Biosciences | 563794 |
| CD3 | Alexa Fluor 532 | UCHT1 | 1:20 | Thermo Fisher Scientific | 58-0038-42 |
| CD11b | Brilliant Violet 785 | ICRF44 | 1:40 | BioLegend | 301346 |
| CD14 | PE | RMO52 | 1:20 | Beckman Coulter | A07764 |
| CD15 | Brilliant Violet 605 | W6D3 | 1:40 | BD Biosciences | 562979 |
| CD16 | PerCP-eFluor 710 | CB16 | 1:66 | Thermo Fisher Scientific | 46-0168-42 |
| CD19 | APC-Fire 810 | HIB19 | 1:100 | BioLegend | 302272 |
| CD33 | Brilliant Violet 510 | P67.6 | 1:100 | BioLegend | 366610 |
| CD45 | APC | J33 | 1:40 | Beckman Coulter | IM2473 |
| CD56 | Brilliant Violet 650 | HCD56 | 1:100 | BioLegend | 318344 |
| CD66b | Alexa Fluor 700 | G10F5 | 1:40 | BioLegend | 305114 |
| HLA-DR | PerCP | L243 | 1:20 | BioLegend | 307628 |
| 6-Sulfo LacNAc (slan) | VioBlue | DD-1 | 1:20 | Miltenyi Biotec | 130-119-868 |
| Stain buffer with BSA | - | - | 1:8.3 | BD Biosciences | 554657 |

BSA = bovine serum albumin, MDSC = myeloid-derived suppressor cell.

**Supplementary Table 5:** Antibodies used for T cell characterization.

| **Antibody** | **Fluorochrome** | **Clone** | **Dilution** | **Manufacturer** | **Article number** |
| --- | --- | --- | --- | --- | --- |
| CD3 | PerCP-Cy5.5 | UCHT1 | 1:40 | BioLegend | 300430 |
| CD4 | APC | SK3 | 1:100 | BioLegend | 344614 |
| CD8 | FITC | SK1 | 1:20 | BD Biosciences | 345772 |
| CD25 | PE | BC96 | 1:66 | BioLegend | 302606 |
| CD45 | PB | J33 | 1:40 | Beckman Coulter | A74763 |
| CD45RO | BV510 | UCHL1 | 1:6.6 | BioLegend | 304246 |

**Supplementary Figures**


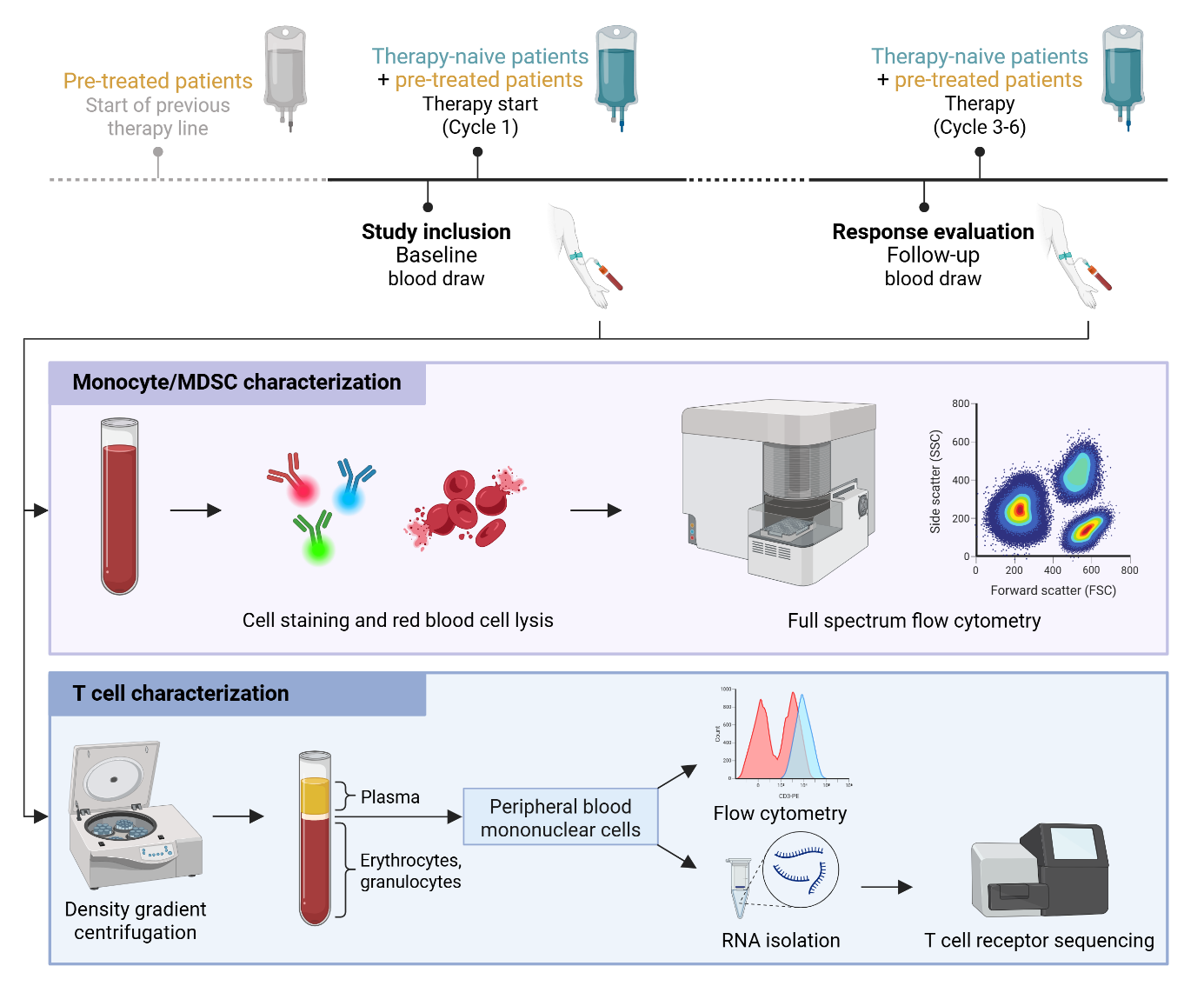


**Supplementary Figure 1:** Overview of patient enrollment, blood collection timepoints, therapy response evaluation, and workflow for the characterization of monocyte and myeloid-derived suppressor cell subsets, T cells, and their receptors.


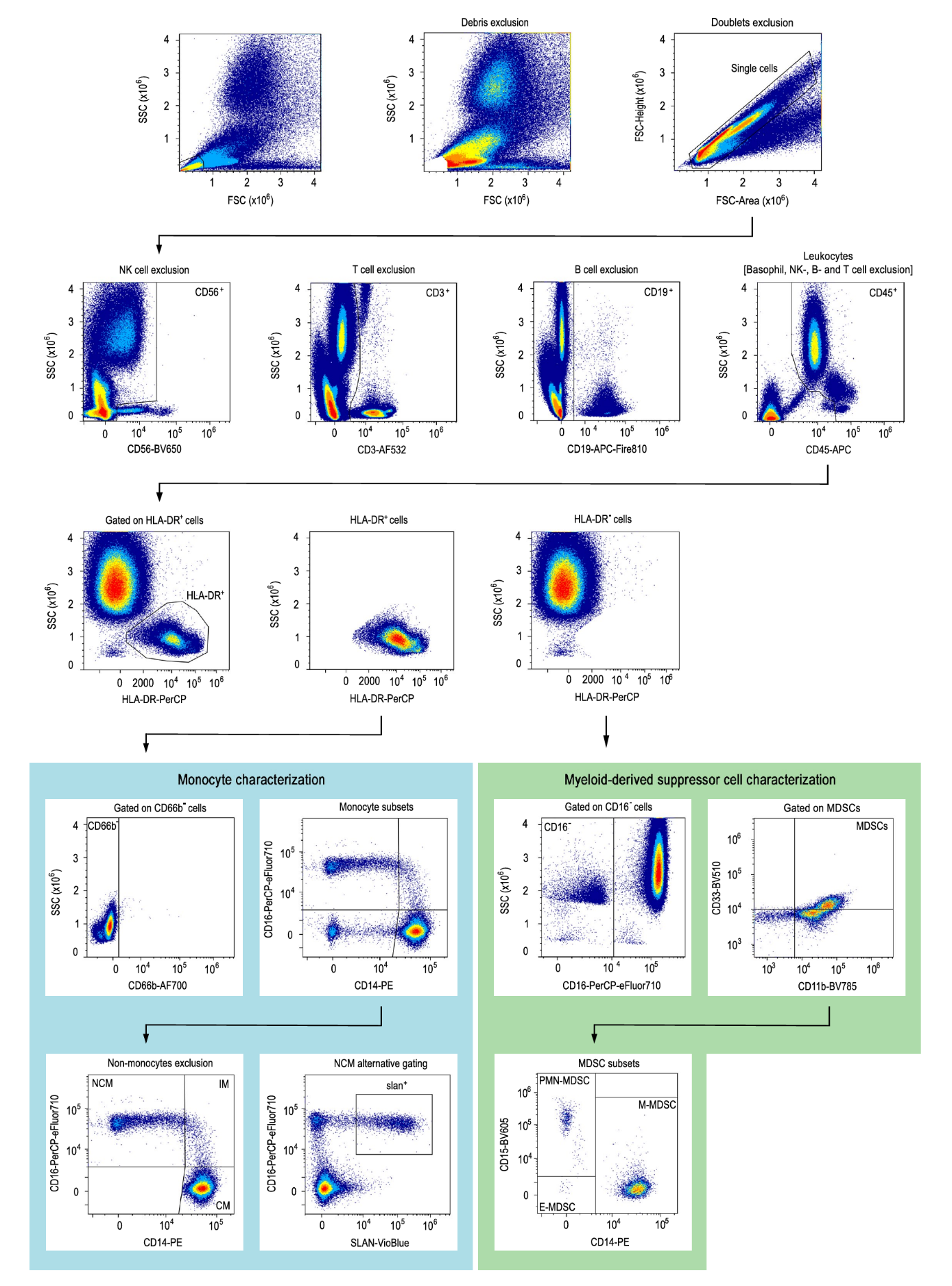


**Supplementary Figure 2:** Gating strategy for peripheral monocyte and MDSC subset characterization from whole blood. Briefly, debris, erythrocytes, doublets, NK cells, and T- and B cells were excluded. Within CD45^+^ leukocytes, basophil granulocytes were excluded, and HLA-DR expression was assessed. Monocytes were characterized within HLA-DR^+^ cells and further divided into subsets, while excluding non-monocytes via Boolean gating. CMs (CD14^++^CD16^−^), IMs (CD14^++^CD16^+^), and NCMs (CD14^+^CD16^++^). MDSC subsets were characterized within HLA-DR^-^ cells after excluding CD16^+^ cells: PMN- (CD14^-^CD15^+^), M- (CD14^+^CD15^-^), and E-MDSCs (CD14^-^CD15^-^). CM = classical monocyte, E-MDSC = early-stage MDSC, FSC = forward scatter, IM = intermediate monocyte, M-MDSC = monocytic MDSC, MDSC = myeloid-derived suppressor cell, NCM = non-classical monocyte, NK cell = natural killer cell, PMN-MDSC = polymorphonuclear MDSC, SSC = side scatter.


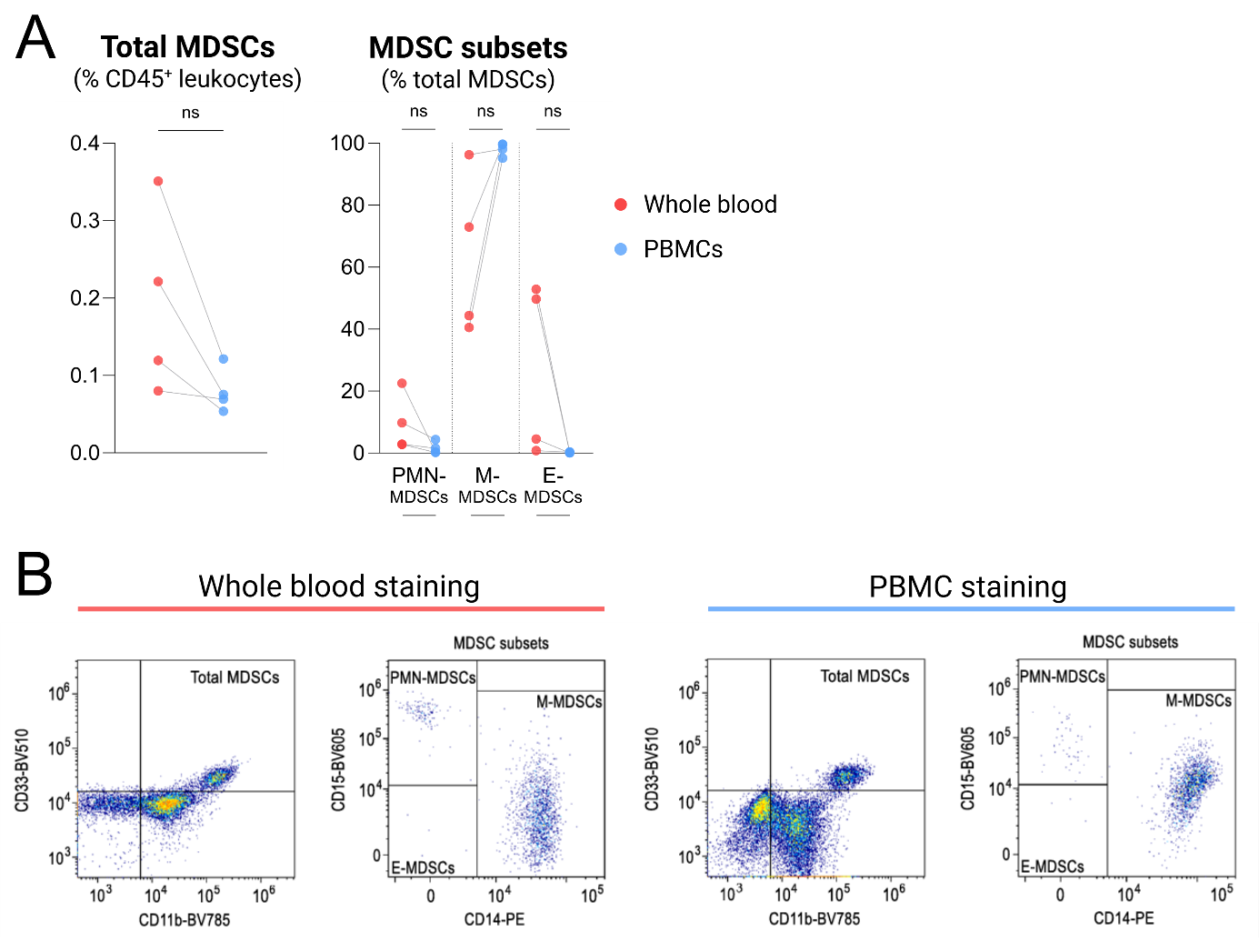
**Supplementary Figure 3:** Paired dot plots displaying differences between whole blood and PBMC staining in (A) total MDSCs and MDSC subsets in four healthy donors. Non-parametric Wilcoxon matched-pairs signed-rank test was used to detect statistical differences. n = 4. (B) Dot plots from one patient illustrating gating differences in total MDSCs and MDSC subsets between whole blood and PBMC staining. Freshly drawn sodium-heparin anticoagulated blood was used. Cells from whole blood and PBMC-derived cells were processed at the same time. E-MDSC = early-stage MDSC, M-MDSC = monocytic MDSC, MDSC = myeloid-derived suppressor cell, PBMC = peripheral blood mononuclear cell, PMN-MDSC = polymorphonuclear MDSC.


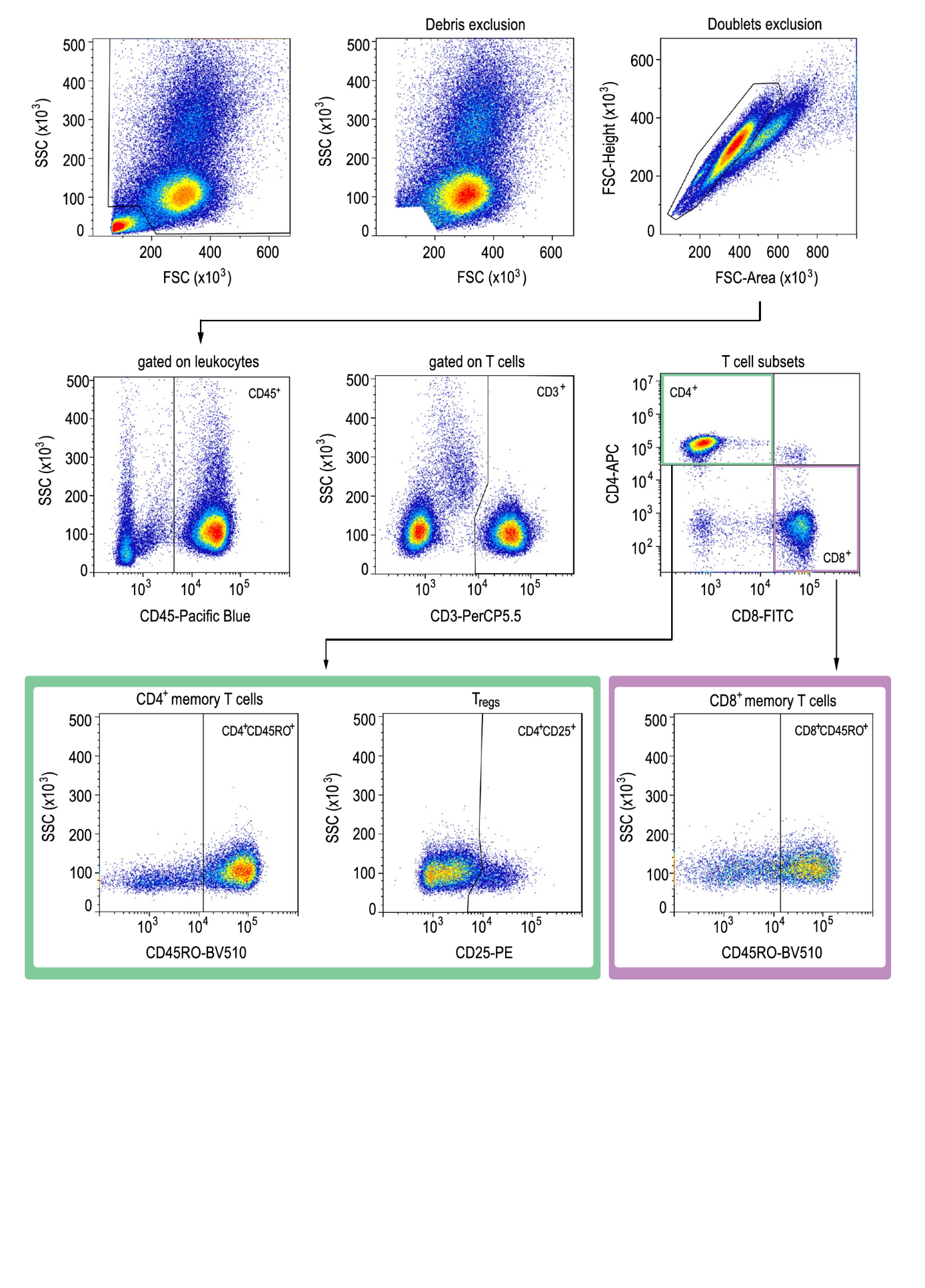


**Supplementary Figure 4:** Gating strategy for peripheral T cell characterization from freshly isolated PBMCs. Briefly, debris and doublets were excluded. CD45^+^ leukocytes were gated from single cells, before CD3^+^ T cells were characterized from leukocytes. Then, CD4^+^ and CD8^+^ T cells were identified within the CD3^+^ T cell population. Further, memory CD4^+^ and CD8^+^ T cells were gated from their respective CD45RO^+^ gate. T_regs_ were characterized by gating on CD25^+^ cells from the CD4^+^ T cell population. FSC = forward scatter, PBMC = peripheral blood mononuclear cell, SSC = side scatter, T_reg_ = regulatory T cell.


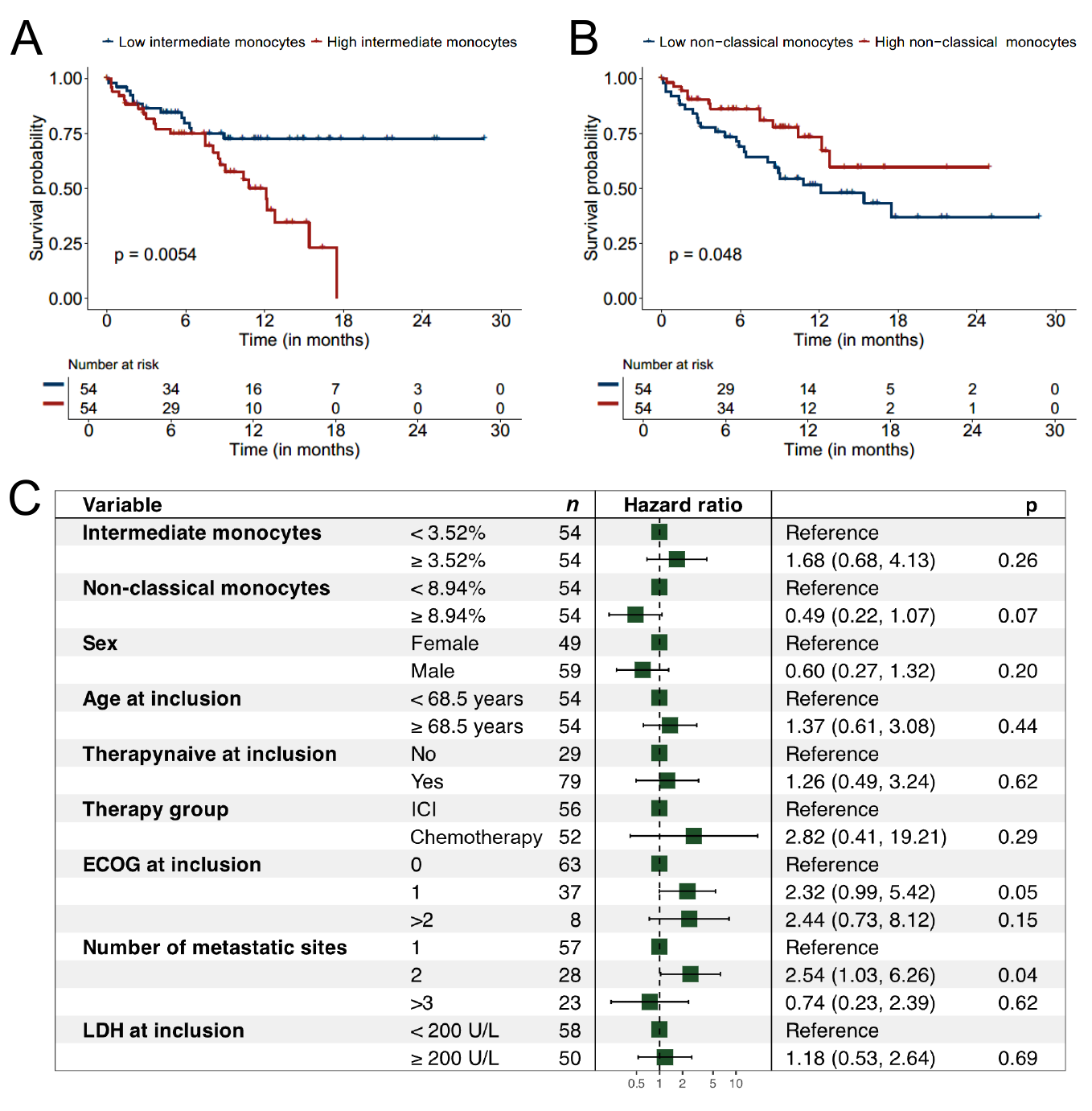
**Supplementary Figure 5:** Kaplan-Meier plots displaying the overall survival probability for patients with high (red line) or low (blue line) (A) intermediate (IMs) and (B) non-classical monocytes (NCMs) at baseline. Variables were dichotomized using the median as cutoff. Survival differences were assessed by log-rank test. (C) Forest plot displaying results from an entity-stratified multivariable Cox proportional hazards regression model assessing the effect of IMs and NCMs on overall survival, while including clinically relevant covariates. Hazard ratios (HRs) with 95% confidence intervals (CIs) are given. HR < 1 improved survival, HR > 1 worse survival. n = 108.


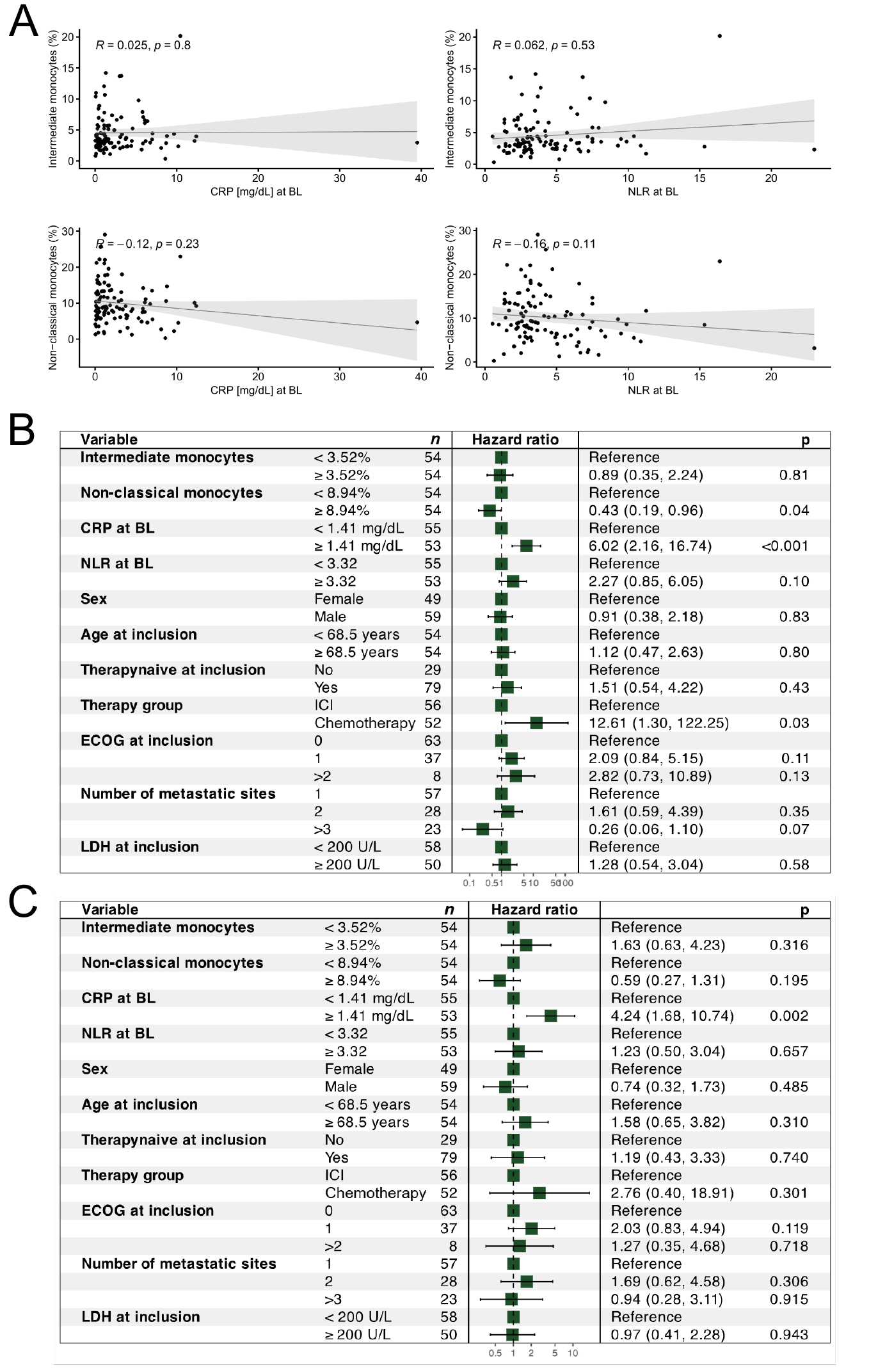


**Supplementary Figure 6:** (A) Dot plots displaying Spearman’s rank correlations between intermediate (IMs), non-classical monocytes (NCMs), C-reactive protein (CRP), and neutrophil-to-lymphocyte (NLR). Forest plots from entity-stratified multivariable Cox proportional hazards regression models assessing the effect of IMs and NCMs on (B) progression-free, and (C) overall survival, adjusted for CRP and NLR as confounders, while including clinically relevant covariates. Hazard ratios (HRs) with 95% confidence intervals (CIs) are given. HR < 1 improved survival, HR > 1 worse survival. n = 108.


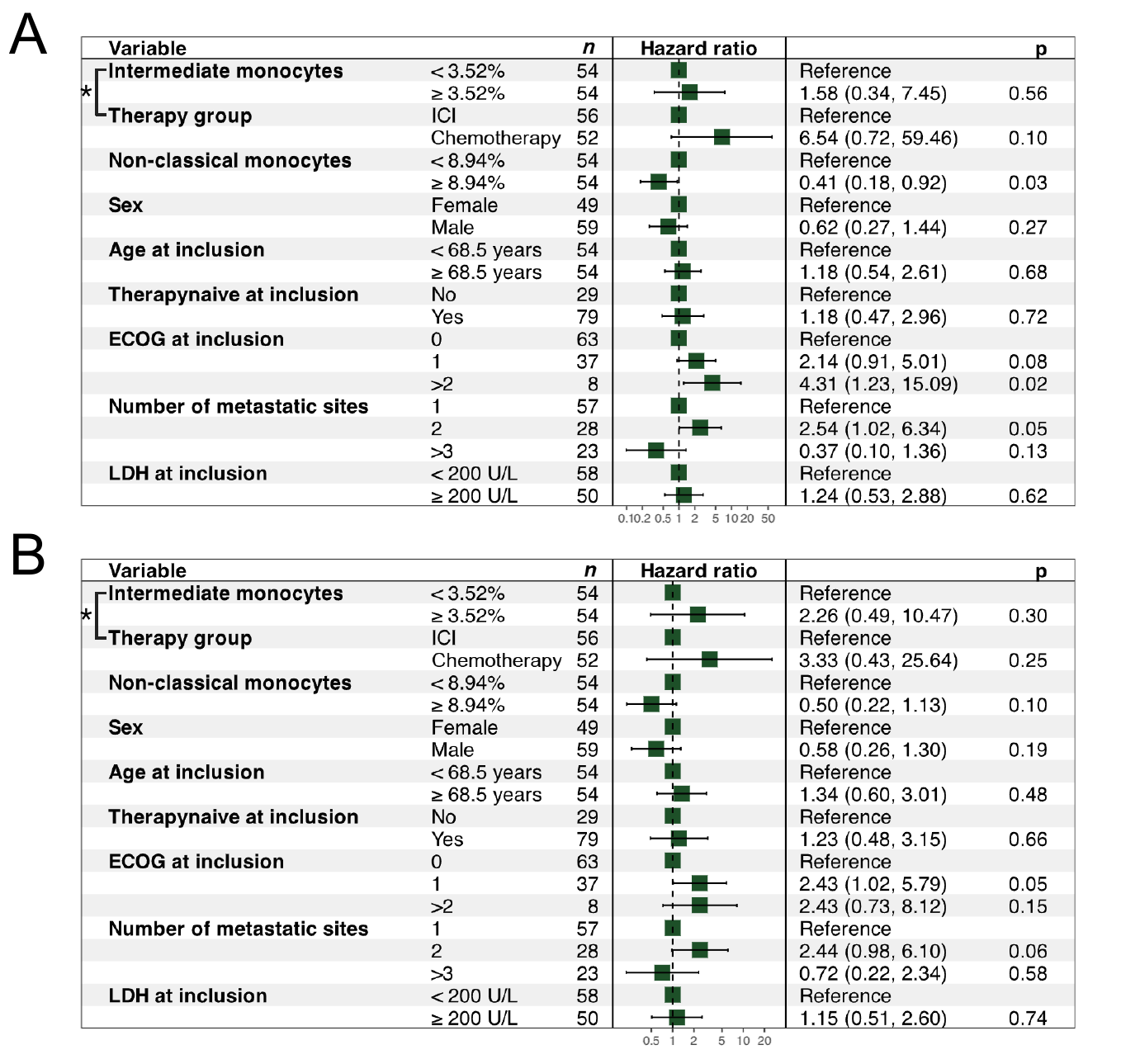


**Supplementary Figure 7:** Forest plots from entity-stratified multivariable Cox proportional hazards regression models assessing the effect of IMs and NCMs on (A) progression-free, and (B) overall survival. Models included interaction terms between IMs and therapy type (indicated as *), as well as clinically relevant covariates. Hazard ratios (HRs) with 95% confidence intervals (CIs) are given. HR < 1 improved survival, HR > 1 worse survival. n = 108.


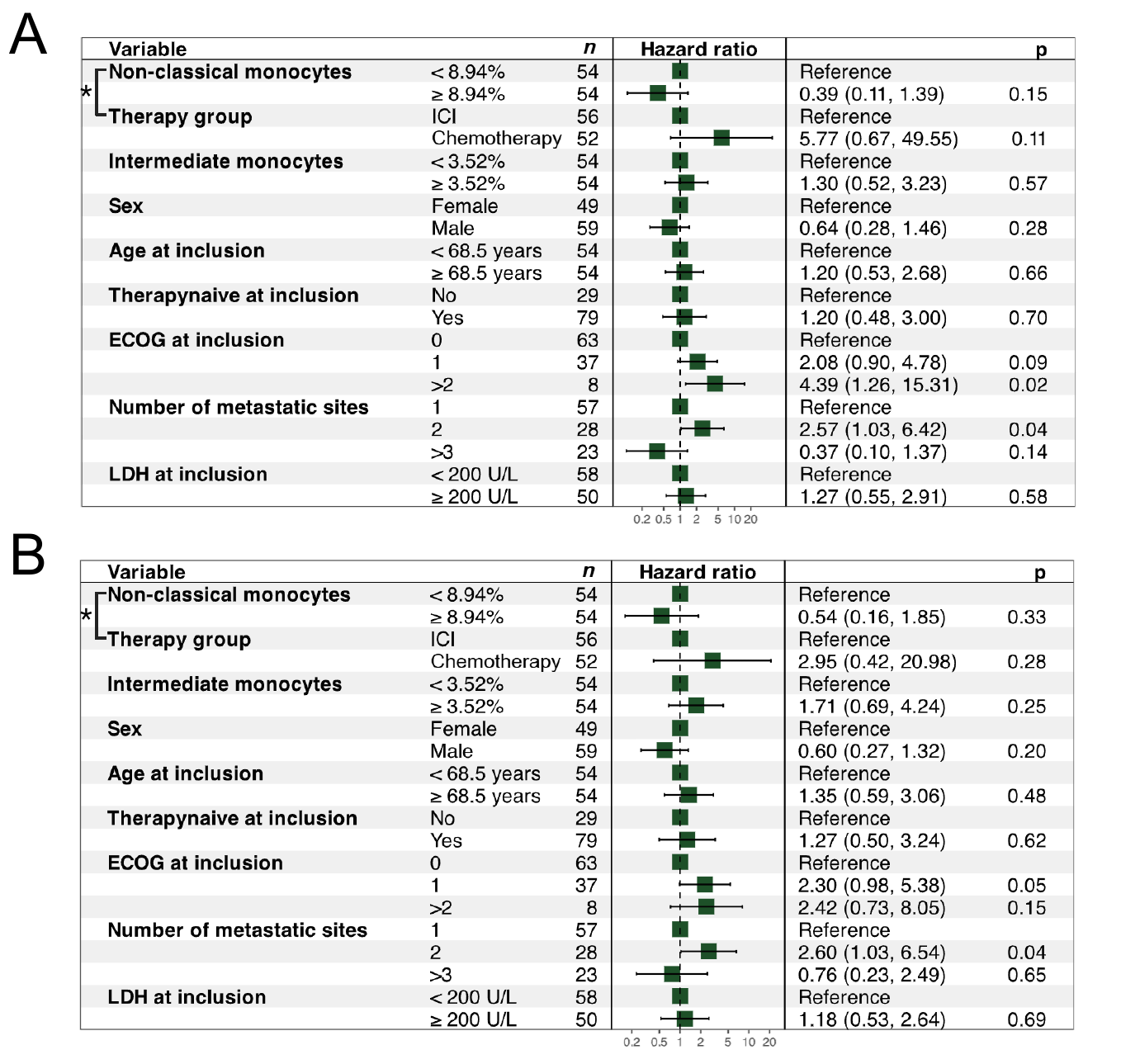


**Supplementary Figure 8:** Forest plots from entity-stratified multivariable Cox proportional hazards regression models assessing the effect of IMs and NCMs on (A) progression-free, and (B) overall survival. Models included interaction terms between NCMs and therapy type (indicated as *), as well as clinically relevant covariates. Hazard ratios (HRs) with 95% confidence intervals (CIs) are given. HR < 1 improved survival, HR > 1 worse survival. n = 108.


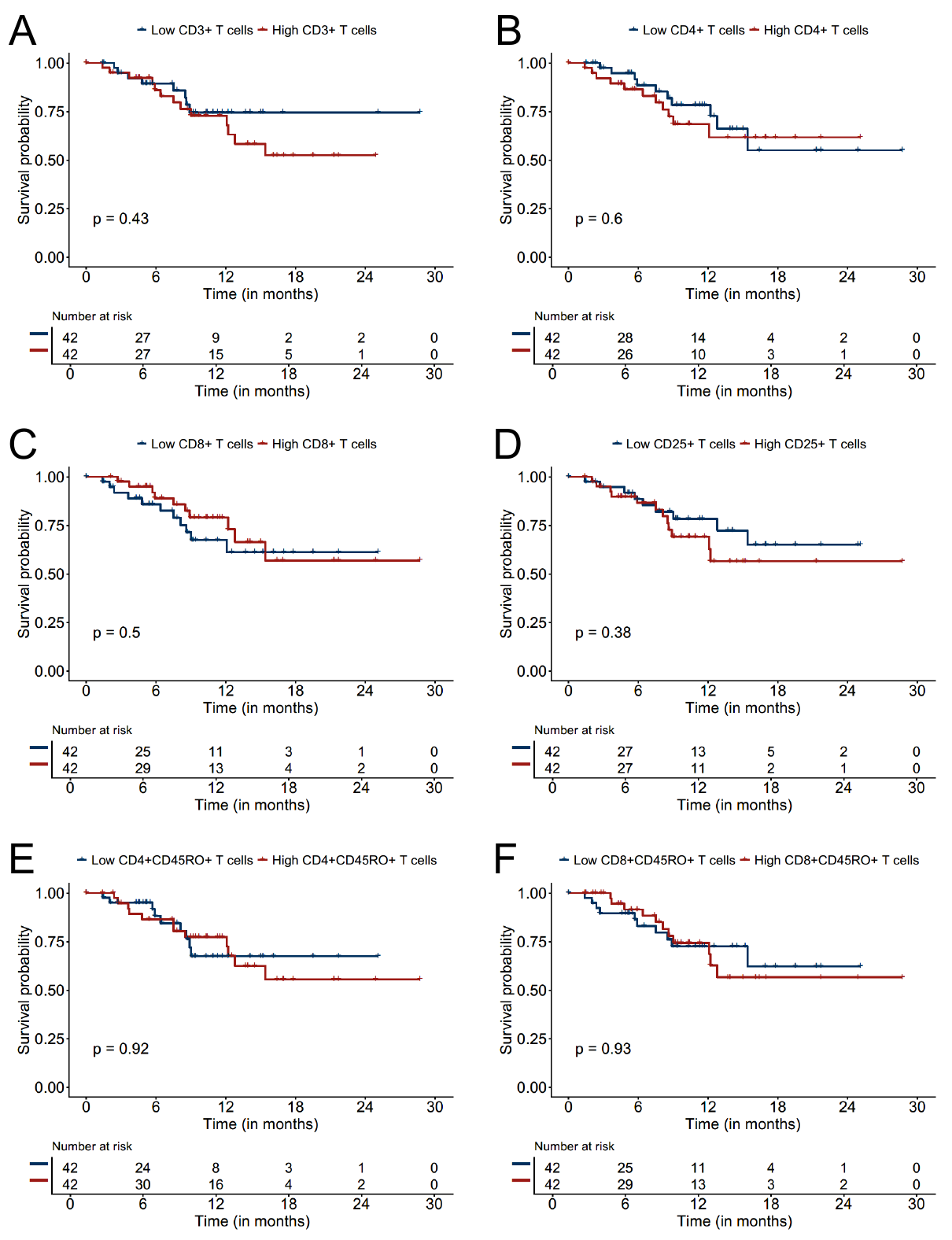


**Supplementary Figure 9:** Kaplan-Meier plots displaying the overall survival probability for patients with high (red line) or low (blue line) (A) CD3^+^, (B) CD4^+^, (C) CD8^+^, (D) CD25^+^, (E) CD4^+^CD45RO^+^, and (F) CD8^+^CD45RO^+^ at baseline. Variables were dichotomized using the median as cutoff. Survival differences were assessed by log-rank test. n = 84.


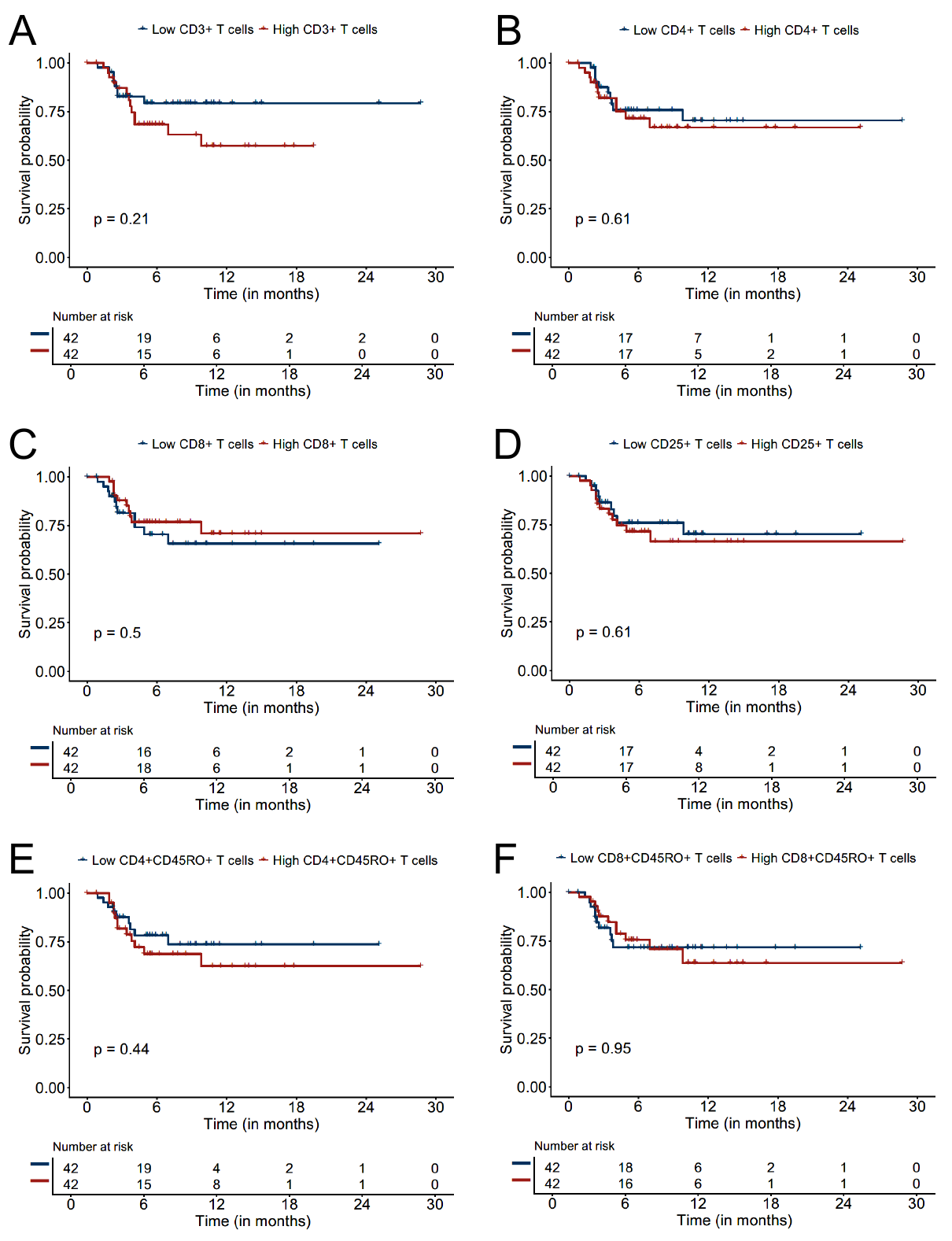


**Supplementary Figure 10:** Kaplan-Meier plots displaying the progression-free survival probability for patients with high (red line) or low (blue line) (A) CD3^+^, (B) CD4^+^, (C) CD8^+^, (D) CD25^+^, (E) CD4^+^CD45RO^+^, and (F) CD8^+^CD45RO^+^ at baseline. Variables were dichotomized using the median as cutoff. Survival differences were assessed by log-rank test. n = 84.


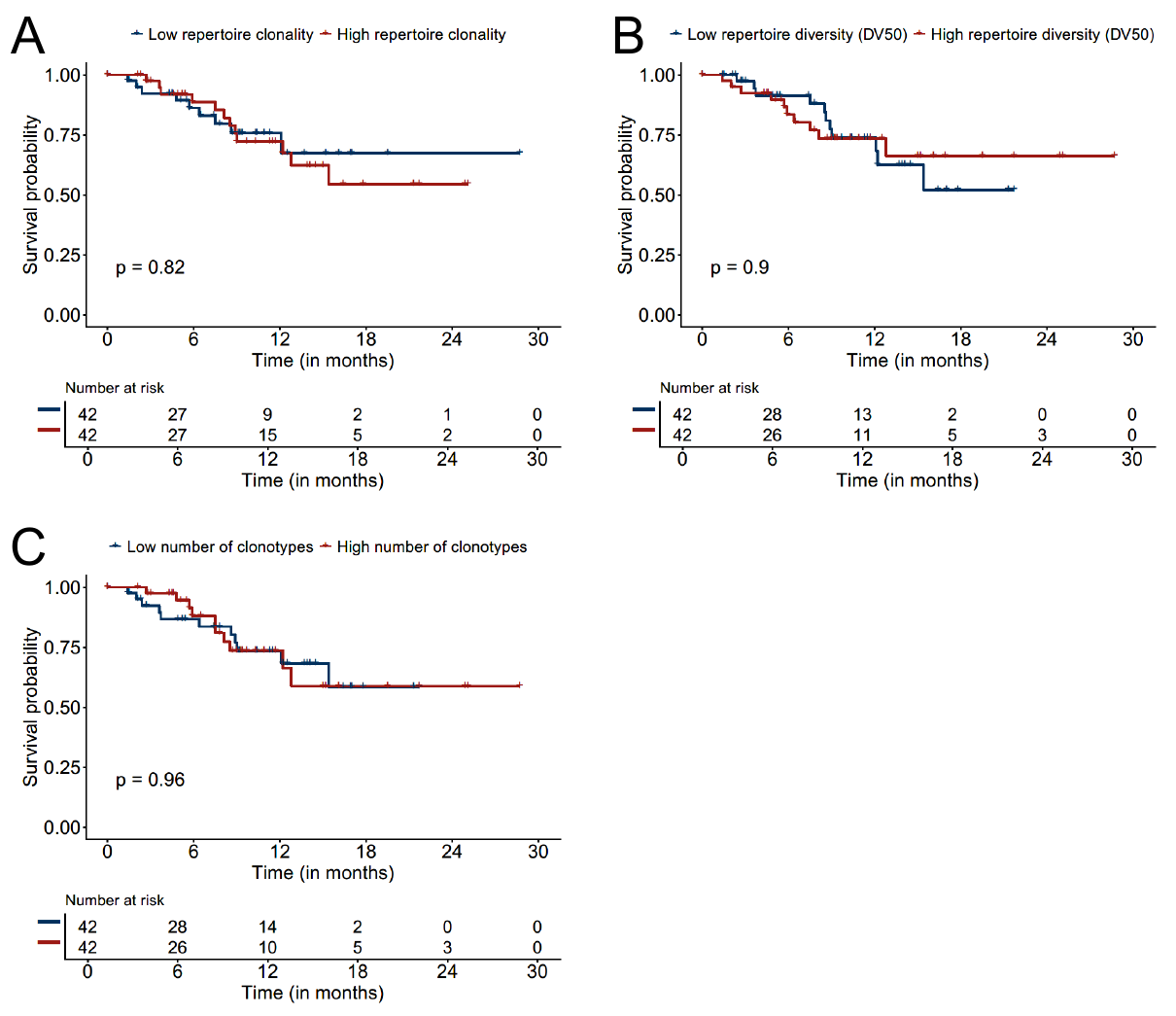


**Supplementary Figure 11:** Kaplan-Meier plots displaying the overall survival probability for patients with high (red line) or low (blue line) TCR repertoire (A) clonality, (B) diversity (DV50), and (C) number of clonotypes at baseline. Variables were dichotomized using the median as cutoff. Survival differences were assessed by log-rank test. n = 84. DV50 = Diversity 50 Index, TCR = T cell receptor.


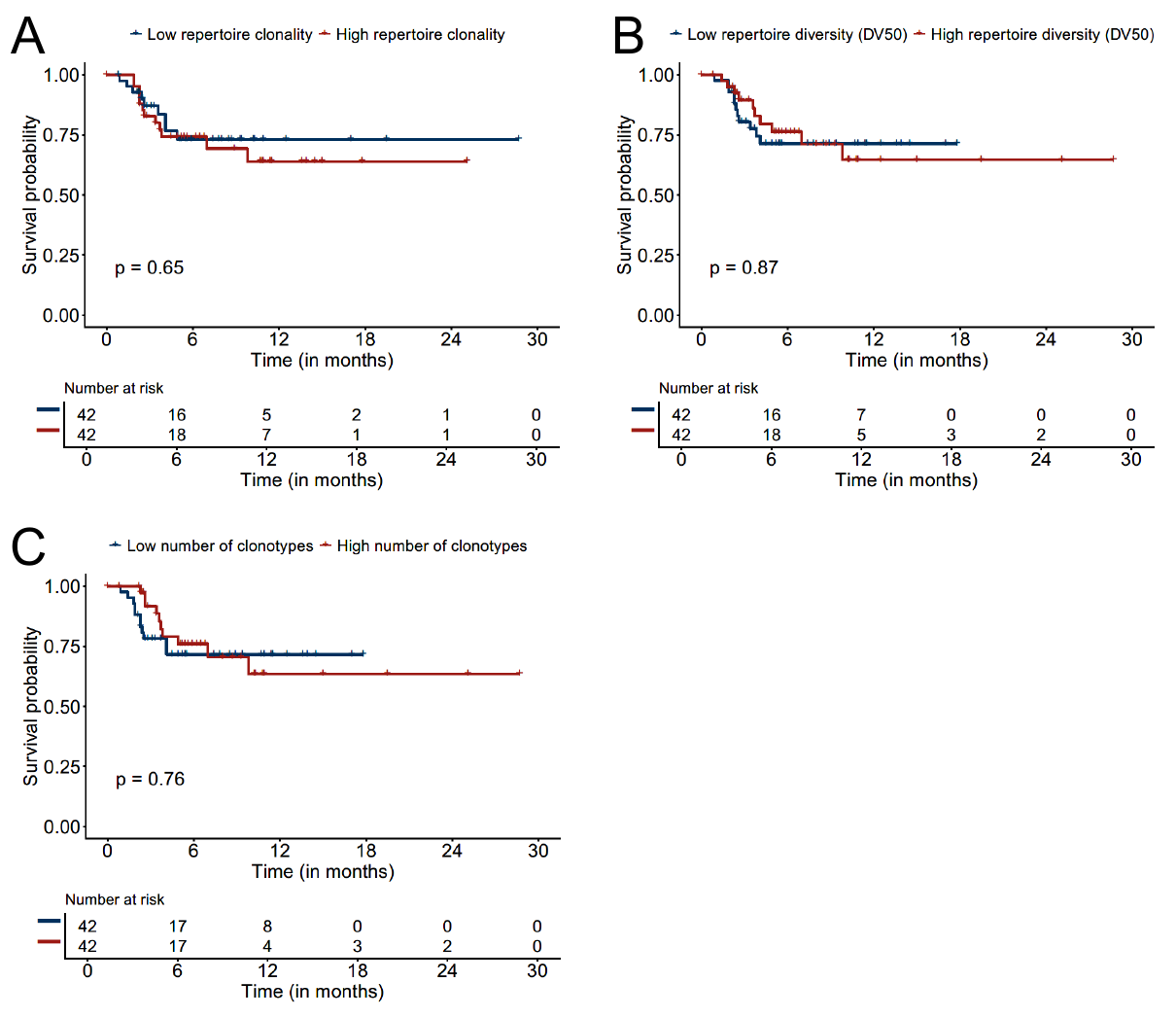


**Supplementary Figure 12**: Kaplan-Meier plots displaying the progression-free survival probability for patients with high (red line) or low (blue line) TCR repertoire (A) clonality, (B) diversity (DV50), and (C) number of clonotypes at baseline. Variables were dichotomized using the median as cutoff. Survival differences were assessed by log-rank test. n = 84. DV50 = Diversity 50 Index, TCR = T cell receptor.


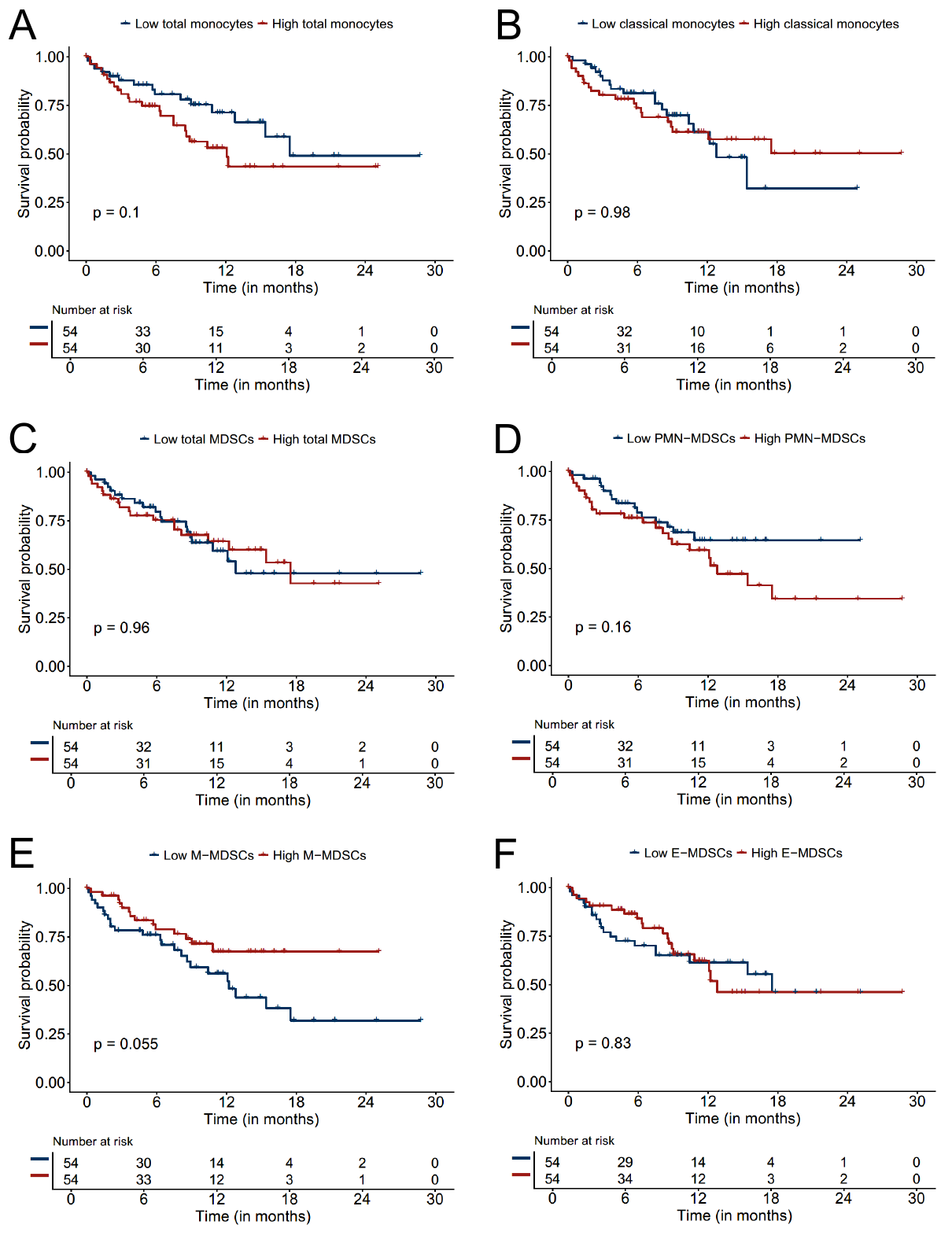


**Supplementary Figure 13:** Kaplan-Meier plots displaying the overall survival probability for patients with high (red line) or low (blue line) (A) total monocytes, (B) classical monocytes, (C) total MDSCs, (D) PMN-MDSCs, (E) M-MDSCs, and (F) E-MDSCs at baseline. Variables were dichotomized using the median as cutoff. Survival differences were assessed by log-rank test. n = 108. E-MDSC = early-stage MDSC, M-MDSC = monocytic MDSC, MDSC = myeloid-derived suppressor cell, PMN-MDSC = polymorphonuclear MDSC.


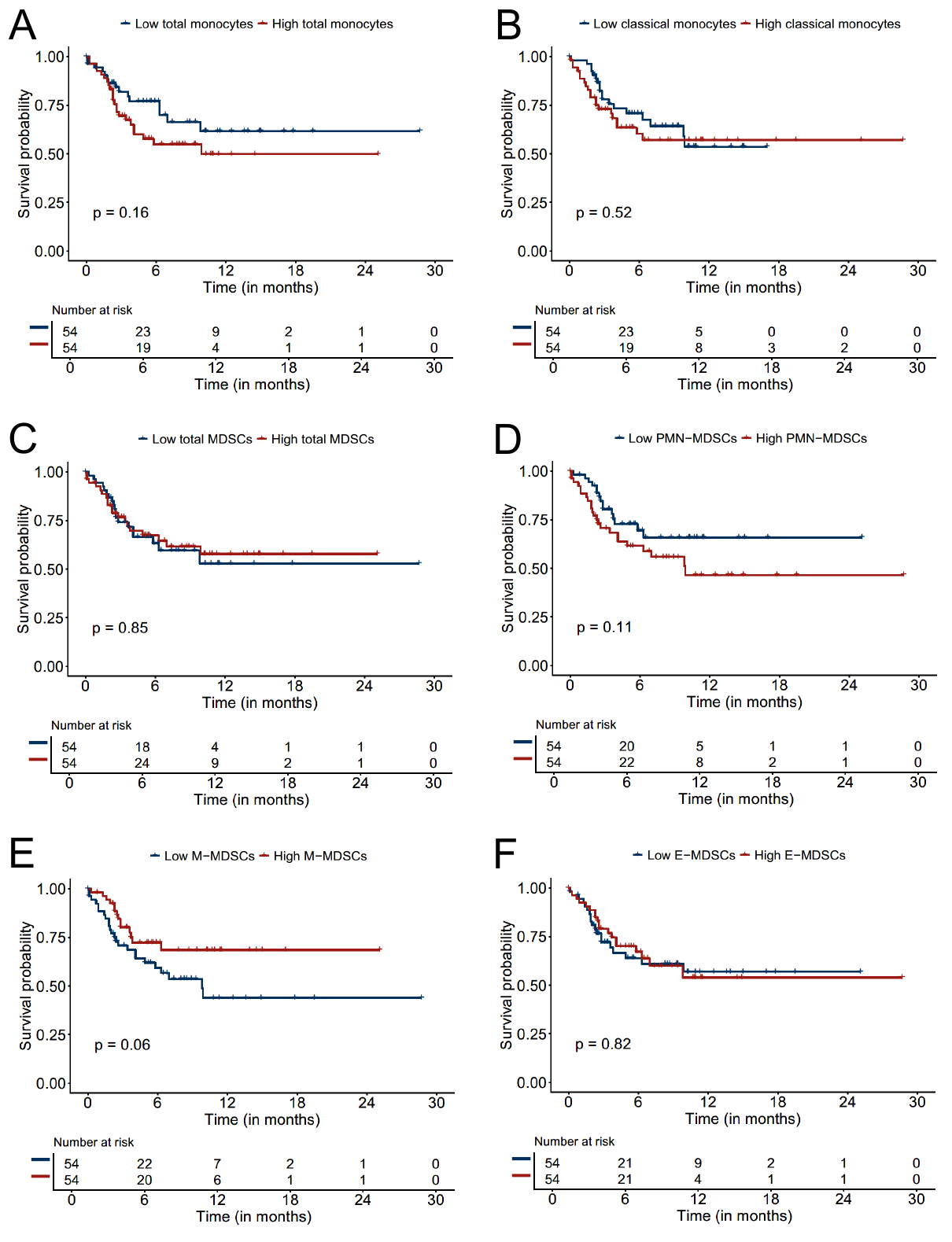


**Supplementary Figure 14:** Kaplan-Meier plots displaying the progression-free survival probability for patients with high (red line) or low (blue line) (A) total monocytes, (B) classical monocytes, (C) total MDSCs, (D) PMN-MDSCs, (E) M-MDSCs, and (F) E-MDSCs at baseline. Variables were dichotomized using the median as cutoff. Survival differences were assessed by log-rank test. n = 108. E-MDSC = early-stage MDSC, M-MDSC = monocytic MDSC, MDSC = myeloid-derived suppressor cell, PMN-MDSC = polymorphonuclear MDSC.


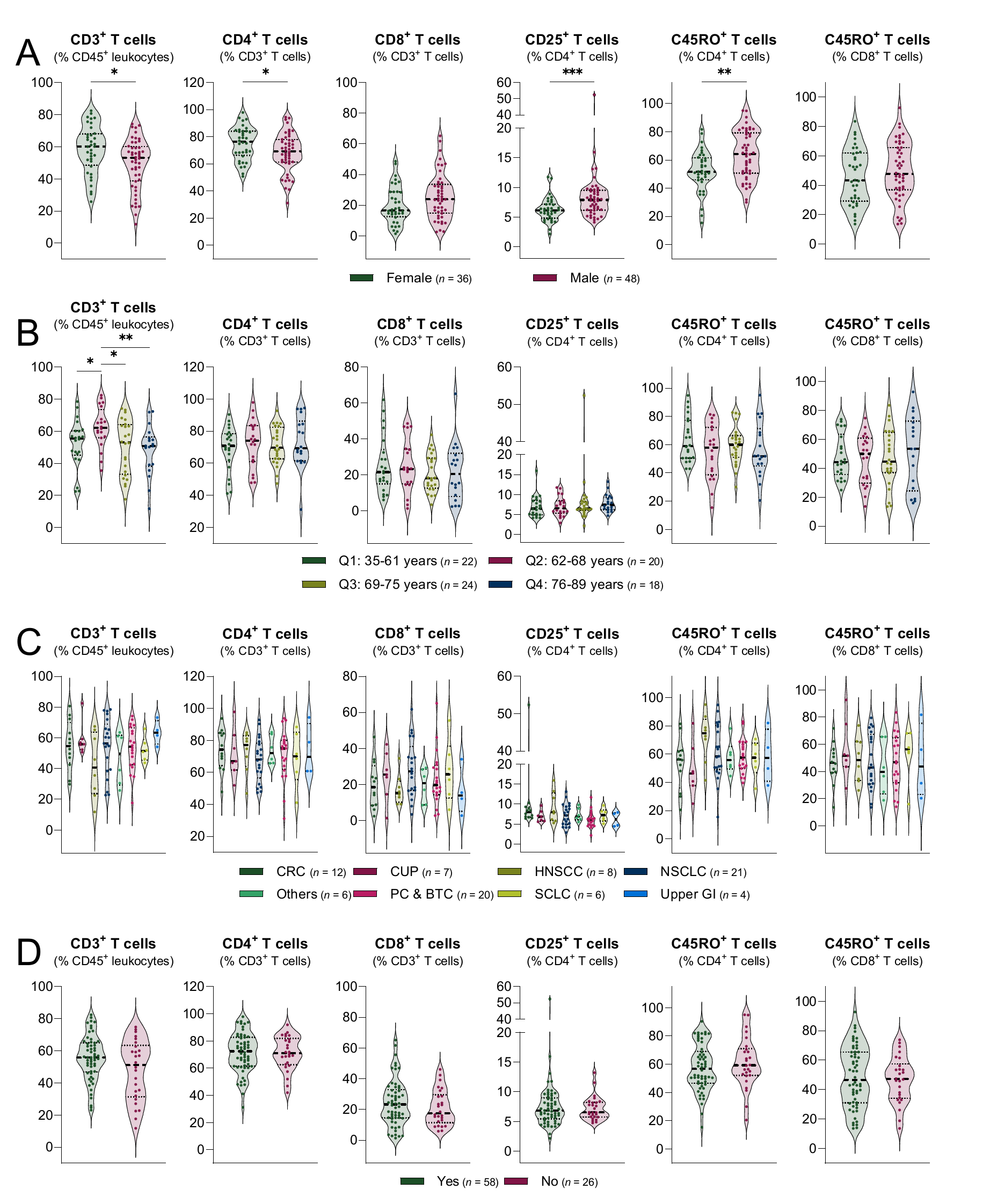


**Supplementary Figure 15:** Violin plots displaying differences in peripheral T cells at baseline stratified by (A) sex, (B) age quartiles, (C) cancer entity, and (D) therapy-naivety at inclusion. Dashed lines represent the median and dotted lines the 25^th^ and 75^th^ percentiles. T cell subsets are displayed as percentage of their respective parent population. Unpaired Mann-Whitney *U* test or Kruskal-Wallis test was used to compare differences between groups using a two-tailed p-value. CRC = colorectal cancer, CUP = cancer of unknown primary, HNSCC = head and neck squamous cell carcinoma, NSCLC = non-small cell lung cancer, PC & BTC = pancreatic cancer and biliary tract cancer, SCLC = small cell lung cancer, Upper GI = upper gastrointestinal tract cancer. ^*^ *P* < 0.05, ^**^ *P* < 0.01, ^***^ *P* < 0.001.


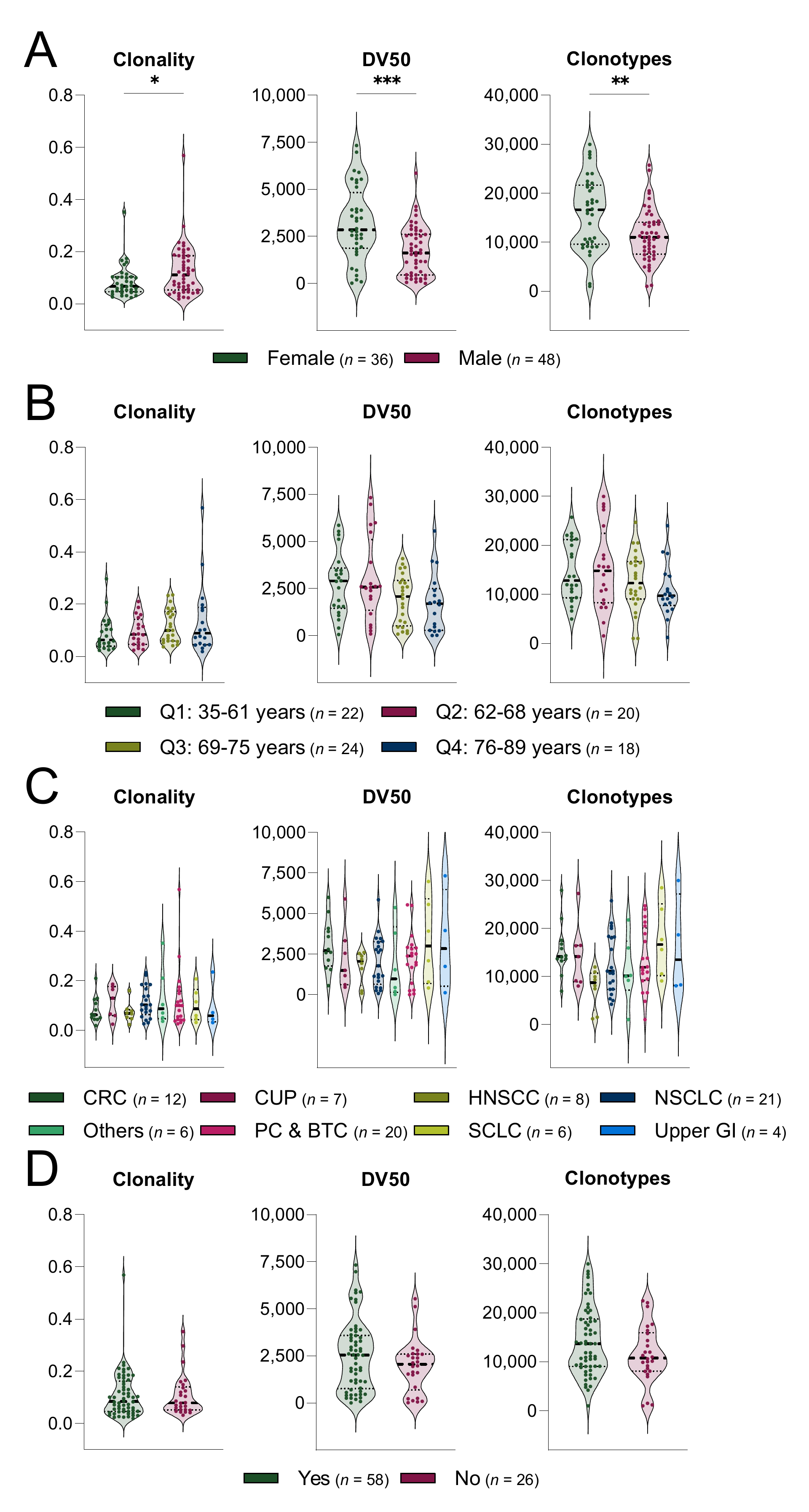


**Supplementary Figure 16:** Violin plots displaying differences in peripheral TCR repertoire at baseline stratified by (A) sex, (B) age quartiles, (C) cancer entity, and (D) therapy-naivety at inclusion. Dashed lines represent the median and dotted lines the 25th and 75th percentiles. Unpaired Mann-Whitney *U* test or Kruskal-Wallis test was used to compare differences between groups using a two-tailed p-value. CRC = colorectal cancer, CUP = cancer of unknown primary, DV50 = Diversity 50 Index, HNSCC = head and neck squamous cell carcinoma, NSCLC = non-small cell lung cancer, PC & BTC = pancreatic cancer and biliary tract cancer, SCLC = small cell lung cancer, Upper GI = upper gastrointestinal tract cancer. ^*^ *P* < 0.05, ^**^ *P* < 0.01, ^***^ *P* < 0.001.


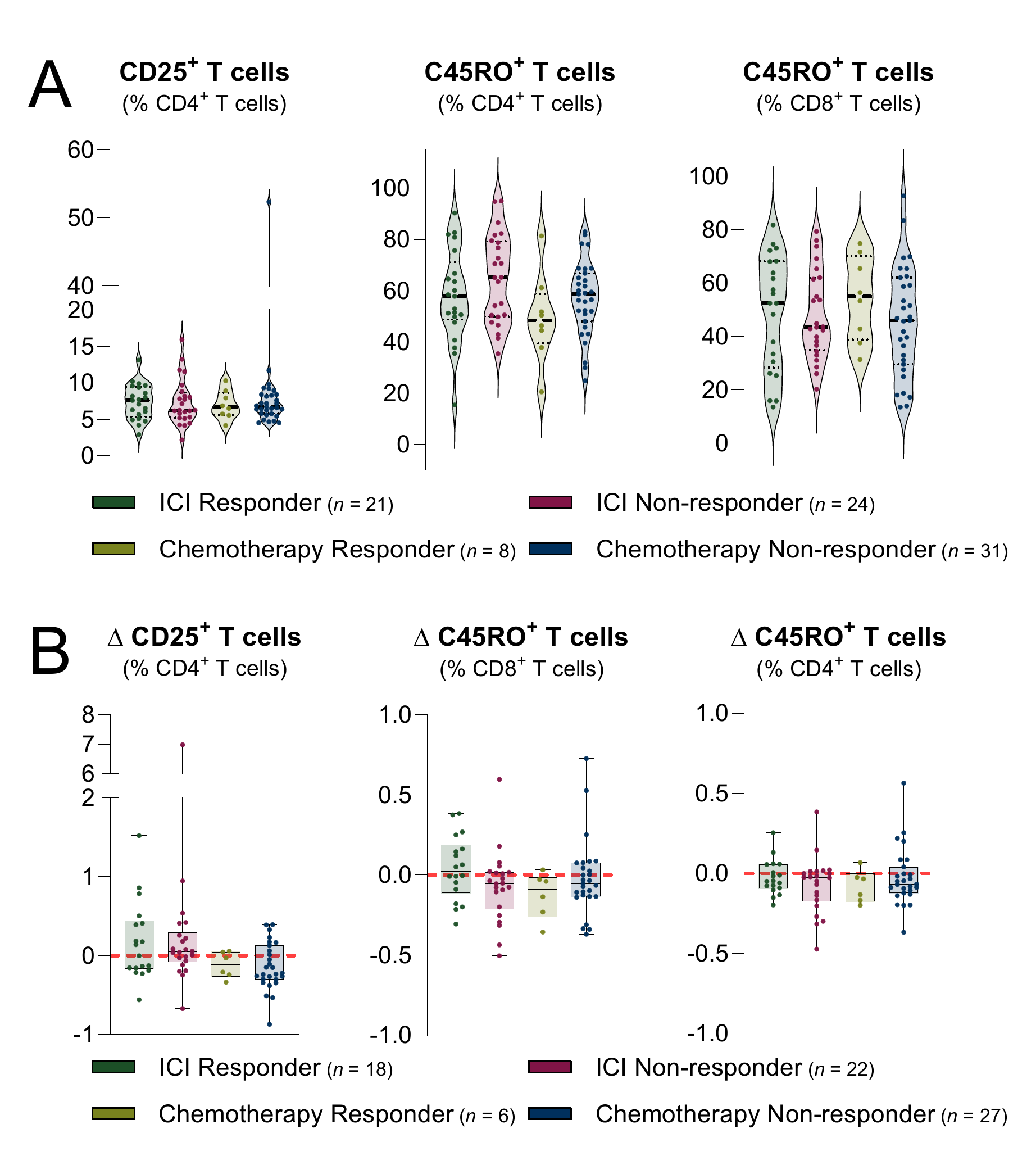


**Supplementary Figure 17:** Violin plots displaying differences at baseline in (A) CD25^+^, CD4^+^CD45RO^+,^ and CD8^+^CD45RO^+^ T cell distribution, and dynamic changes between baseline and 1^st^ follow-up as delta in (B) between ICI and chemotherapy responders and non-responders. Dashed lines represent the median and dotted lines the 25^th^ and 75^th^ percentiles. T cell subsets are displayed as percentage of their respective parent population. Unpaired Mann-Whitney *U* test was used to compare differences between groups using a two-tailed p-value. ICI = immune checkpoint inhibition.


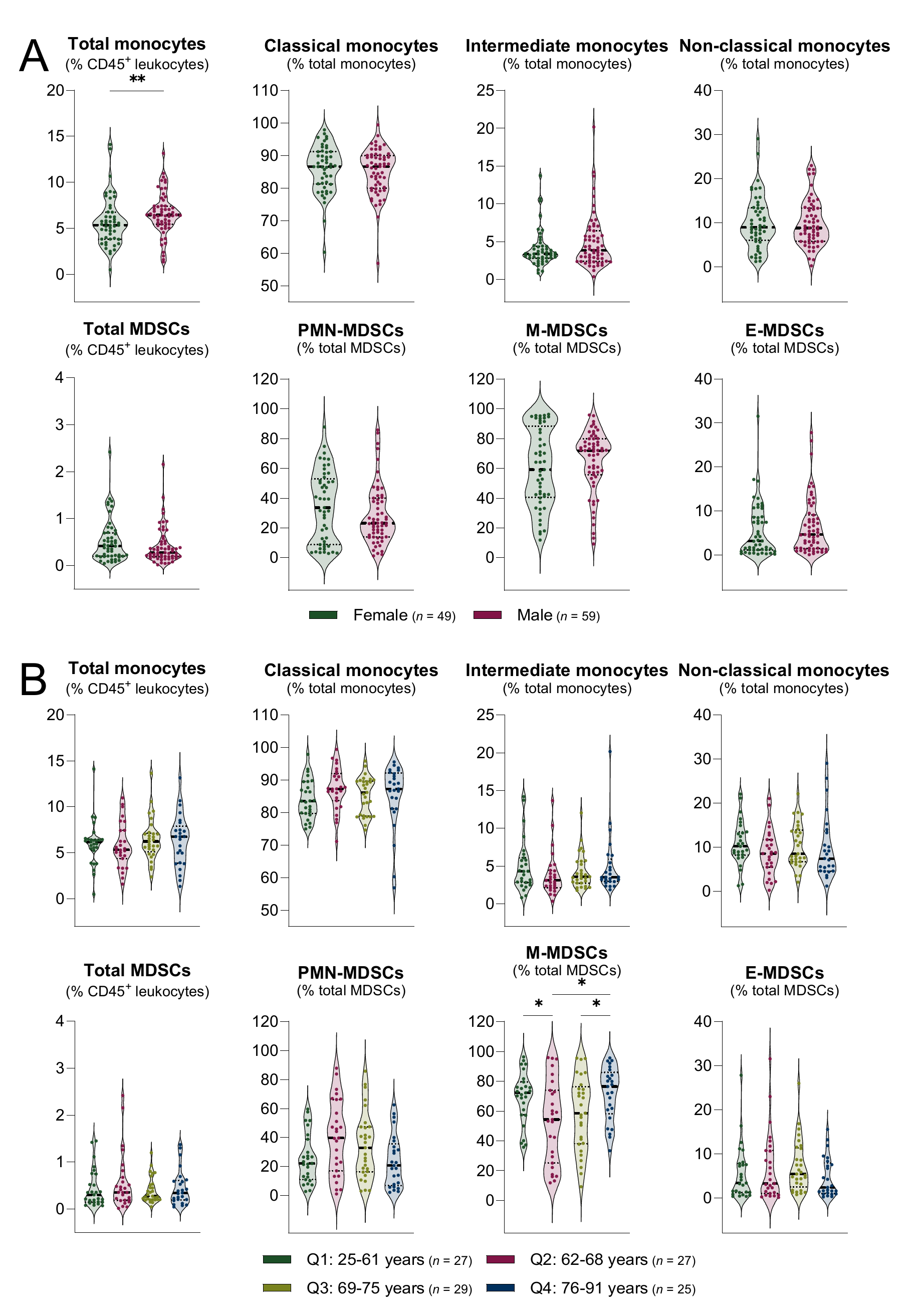

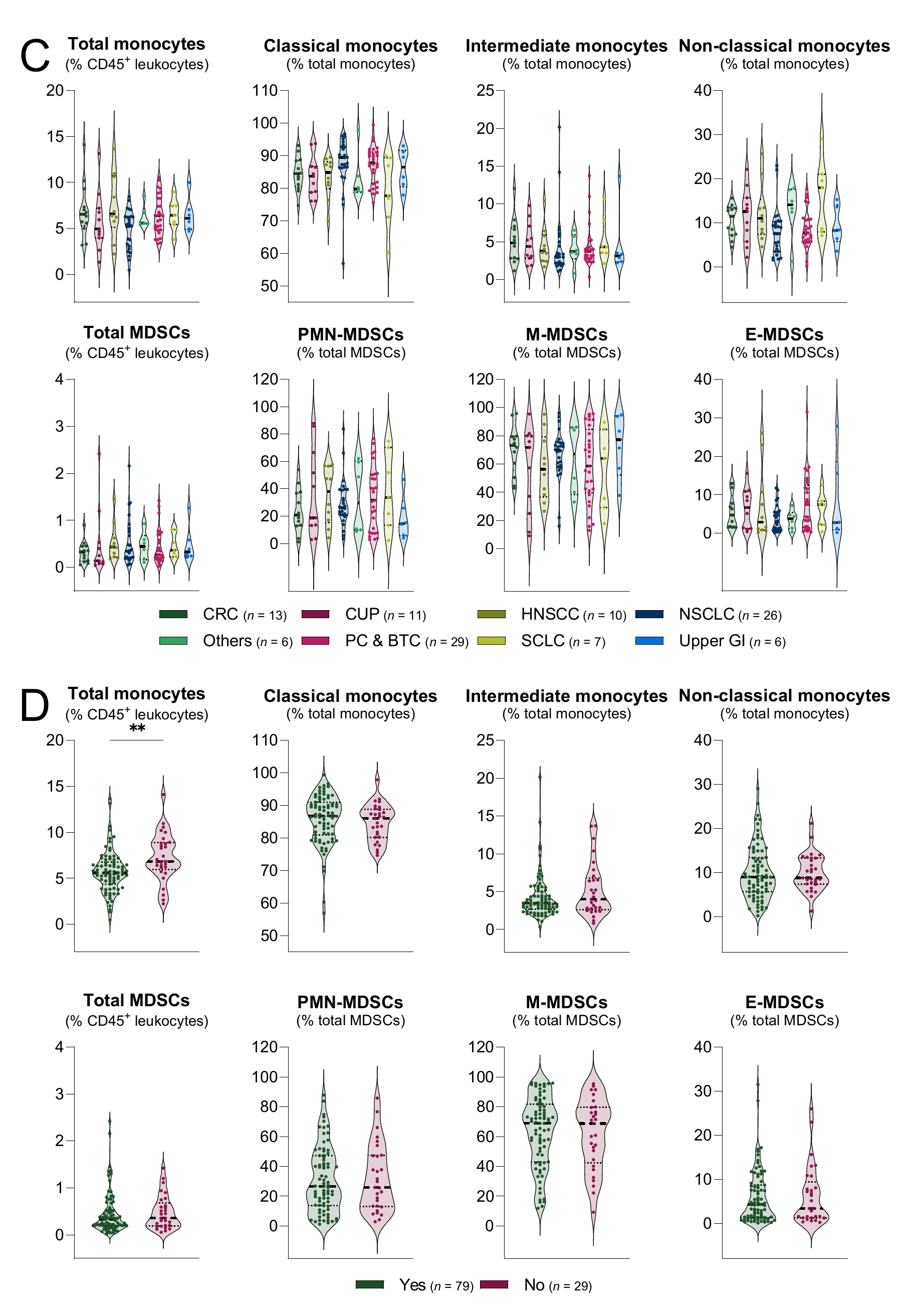


**Supplementary Figure 18:** Violin plots displaying differences in peripheral monocyte and MDSC subsets at baseline stratified by (A) sex, (B) age quartiles, (C) cancer entity, and (D) therapy-naivety at inclusion. Dashed lines represent the median and dotted lines the 25^th^ and 75^th^ percentiles. Monocyte and MDSC subsets are displayed as percentage of total monocytes or total MDSCs, respectively. Unpaired Mann-Whitney *U* test or Kruskal-Wallis test was used to compare differences between groups using a two-tailed p-value. CRC = colorectal cancer, CUP = cancer of unknown primary, E-MDSC = early-stage MDSC, HNSCC = head and neck squamous cell carcinoma, M-MDSC = monocytic MDSC, MDSC = myeloid-derived suppressor cell, NSCLC = non-small cell lung cancer, PBMC = peripheral blood mononuclear cell, PC & BTC = pancreatic cancer and biliary tract cancer, PMN-MDSC = polymorphonuclear MDSC, SCLC = small cell lung cancer, Upper GI = upper gastrointestinal tract cancer. ^*^ *P* < 0.05, ^**^ *P* < 0.01.


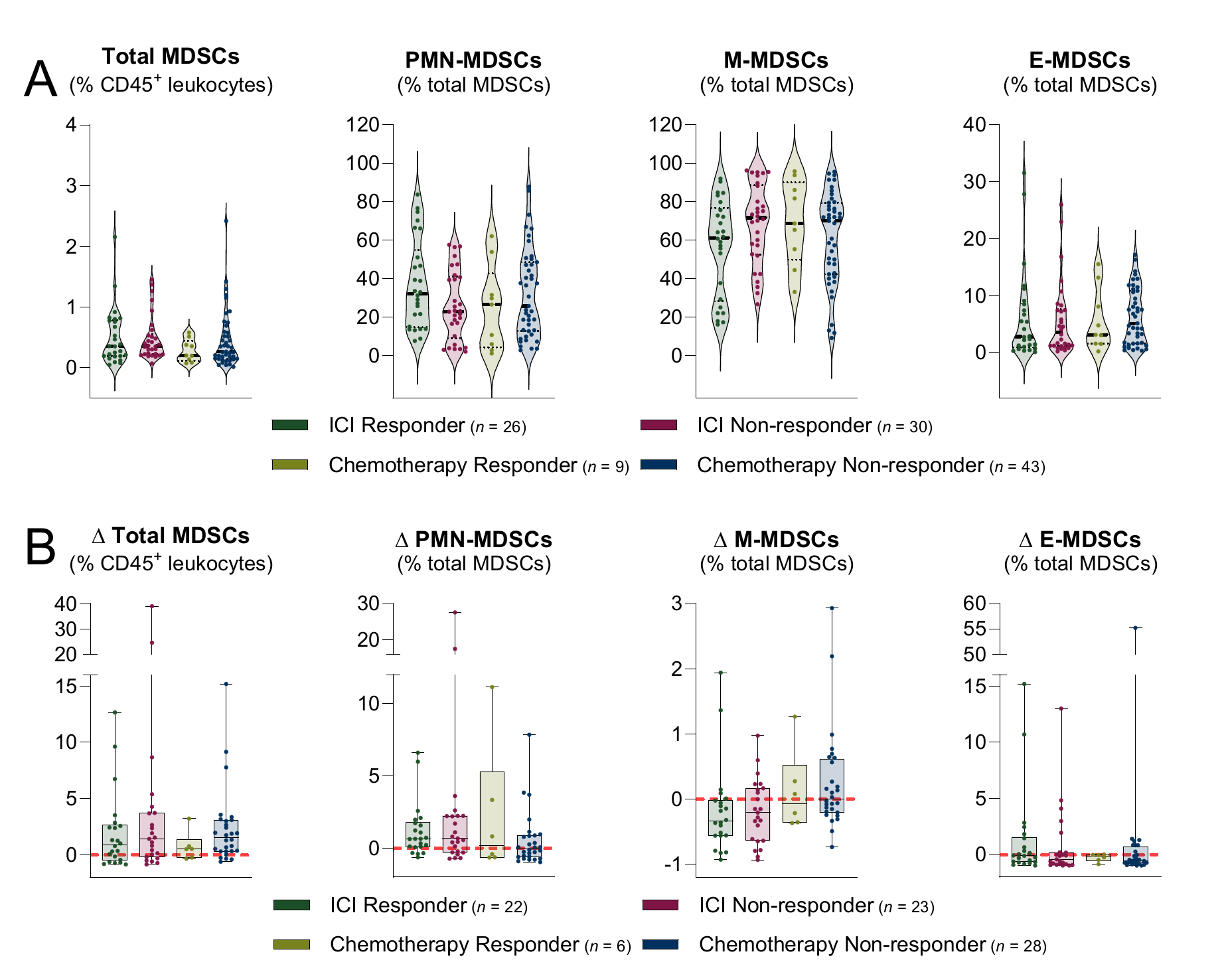


**Supplementary Figure 19:** Violin plots displaying differences at baseline in (A) total MDSCs and their subsets, and dynamic changes between baseline and 1^st^ follow-up as delta in (B) between ICI and chemotherapy responders and non-responders. Dashed lines represent the median and dotted lines the 25^th^ and 75^th^ percentiles. MDSC subsets are displayed as percentage of total MDSCs. An unpaired Mann-Whitney *U* test was used to compare differences between groups using a two-tailed p-value. E-MDSC = early-stage MDSC, ICI = immune checkpoint inhibition, M-MDSC = monocytic MDSC, MDSC = myeloid-derived suppressor cell, PMN-MDSC = polymorphonuclear MDSC.
